# miR-369-3p Modulates Intestinal Inflammatory Response via BRCC3/NLRP3 Inflammasome Axis

**DOI:** 10.3390/cells12172184

**Published:** 2023-08-31

**Authors:** Viviana Scalavino, Emanuele Piccinno, Anna Maria Valentini, Nicolò Schena, Raffaele Armentano, Gianluigi Giannelli, Grazia Serino

**Affiliations:** National Institute of Gastroenterology S. De Bellis, IRCCS Research Hospital, Via Turi 27, 70013 Castellana Grotte, BA, Italy; viviana.scalavino@irccsdebellis.it (V.S.); emanuele.piccinno@irccsdebellis.it (E.P.); am.valentini@irccsdebellis.it (A.M.V.); nicolo.schena@irccsdebellis.it (N.S.); raffaele.armentano@irccsdebellis.it (R.A.); gianluigi.giannelli@irccsdebellis.it (G.G.)

**Keywords:** microRNA, NLRP3, inflammasome, BRCC3, miR-369-3p

## Abstract

Inflammasomes are multiprotein complexes expressed by immune cells in response to distinct stimuli that trigger inflammatory responses and the release of pro-inflammatory cytokines. Evidence suggests a different role of inflammasome NLRP3 in IBD. NLRP3 inflammasome activation can be controlled by post-translational modifications such as ubiquitination through BRCC3. The aim of this study was to investigate the effect of miR-369-3p on the expression and activation of NLRP3 inflammasomes via BRCC3 regulation. After bioinformatics prediction of Brcc3 as a gene target of miR-369-3p, in vitro, we validated its modulation in bone marrow-derived macrophages (BMDM). The increase in miR-369-3p significantly reduced BRCC3 gene and protein expression. This modulation, in turn, reduced the expression of NLRP3 and blocked the recruitment of ASC adaptor protein by NLRP3. As a result, miR-369-3p reduced the activity of Caspase-1 by the inflammasome, decreasing the cleavage of pro-IL-1β and pro-IL-18. These results support a novel mechanism that seems to act on post-translational modification of NLRP3 inflammasome activation by BRCC3. This may be an interesting new target in the personalized treatment of inflammatory disorders, including IBD.

## 1. Introduction

Inflammatory bowel disease (IBD) is a group of disorders of the gastrointestinal tract, including Crohn’s disease (CD) and ulcerative colitis (UC), characterized by chronic and relapsing intestinal inflammation [1]. They are multifactorial disorders in which an altered and chronic activation of the immune system leads to a persistent inflammatory state that promotes the onset and maintenance of disease [2,3].

Innate immunity is the first line of defense against microorganisms and other harmful agents. Immune cells, such as macrophages, release cytokines and chemokines that are critical in the initiation, maintenance, and resolution of inflammation. These mechanisms affect the intestinal epithelial cells, promoting the injuries to the intestinal tissue that are characteristic of IBD [4].

Inflammasomes are cytosolic multiprotein complexes assembled by immune cells in response to distinct stimuli associated to stress or infection, that mediate the activation and release of pro-inflammatory cytokines triggering the inflammatory responses [5,6]. In non-immune cells, including adult stem cells, it has been demonstrated that the activation of NLRP3 inflammasomes was able to regulate the activation of inflammasomes in macrophages [7]. One study also reported the expression of inflammasome components and their functions in stem cells [8]. Generally, the inflammasomes consist of a sensor protein, constituted by nucleotide-binding oligomerization- (NOD-) receptors (NLR); a member of pattern recognition receptors (PRR), an apoptosis-associated adaptor protein; a speck-like protein containing a caspase recruitment domain, namely apoptosis-associated speck-like protein containing a CARD (ASC); and the effector pro-caspase-1 [9]. The sensor protein is located within the cytoplasm and is able to recognize molecular models associated with pathogens/damage (PAMP/DAMP). When activated, members of inflammasomes are assembled and oligomerized, causing the cleavage of pro-caspase-1 in its activated form. The activated caspase-1 (CASP1) has a crucial role in the subsequent inflammatory response leading to the cleavage of interleukin (IL-) 1β and 18 and the initiation of an inflammatory form of cell death mediated by pyroptosis [9,10]. Different inflammasomes have been identified [9]. Among them, NLRP3 is the inflammasome that plays a key role in the gut, regulating immune responses and intestinal homeostasis [11].

The activation of inflammasome NLRP3 involves a two-signal model. The first signal is induced by microbial components such as Lipopolysaccharide (LPS) or endogenous inflammatory cytokines such as Tumor Necrosis Factor alpha (TNF-α). The second signal is triggered by different stimuli that promote the activation and assembly of NLRP3 inflammasomes, such as ATP, pore-forming toxins, viral RNA, and particulate matter [10,12]. In addition to the mechanism described above, which depends on the type and duration of the stimuli, NLRP3 activation is controlled by different combinations of post-translational modifications (PTM) such as phosphorylation and ubiquitination [10,13]. The ubiquitin system has a key role in signaling networks originating from the PRR receptors [14]. It is emerging as an interesting area that may serve to identify novel therapeutic targets to regulate NLRP3 inflammasome activation. In fact, ubiquitination and deubiquitination are crucial steps in the degradation or activation of NLRP3 inflammasomes [13,15,16,17,18]. BRCA1-/BRCA2-containing complex 3 (BRCC3, also known as BRCC36) is a deubiquitinating enzyme that promotes the activation of NLRP3 inflammasomes through the deubiquitination of NLRP3 protein at the post-translational level [16].

microRNAs (miRNAs) are small molecules of non-coding RNA, with an average length of 22 nucleotides, that transcriptionally modulate the expression of several target genes [19]. miRNAs have a relevant role as regulators of several pathways involved in immune responses in normal as well as in pathological conditions [20,21]. We previously proved that miR-369-3p was able to ameliorate inflammatory responses, showing it to be a good candidate for the treatment of chronic inflammation. In fact, we demonstrated that miR-369-3p modulated the release of pro-inflammatory cytokines and the activation of NF-kB signaling pathways through the regulation of C/EBP-β and NOS2 expression [22,23]. Moreover, it was also able to decrease the expression of PSMB9/LMP2, an immunoproteasome subunit, modulating the formation of the complex itself [24].

In the present study, we aimed to investigate the effect of miR-369-3p on the expression and activation of the NLRP3 inflammasome. We demonstrated that under inflammatory conditions, miR-369-3p is able to decrease the expression of Brcc3 in macrophages, modulating the deubiquitination of NLRP3, and hence the assembly and activation of the complex. This reduction consequently decreases the activation of caspase-1 and therefore the expression of pro-inflammatory cytokines Il-1β and Il-18. These results further underline the anti-inflammatory properties of miR-369-3p.

## 2. Materials and Methods

### 2.1. Cell Cultures

Mouse bone marrow-derived macrophages (BMDM) from WT C57BL/6 mice was gifted by Dr Roberto Negro. Cells were grown at 37 °C in a humidified atmosphere with 5% CO_2_ using culture media composed by Dulbecco’s Modified Eagle Medium (DMEM, Thermo Fisher Scientific, Waltham, MA, USA), 10% heat-inactivated Fetal Bovine Serum (FBS, Thermo Fisher Scientific, Waltham, MA, USA), 1% 10,000 µg/mL streptomycin and 10,000 U/mL penicillin (Thermo Fisher Scientific, Waltham, MA, USA), 1% 1M HEPES (Sigma-Aldrich, St. Louis, MO, USA) and 1% 100 mM sodium pyruvate (Sigma-Aldrich, St. Louis, MO, USA).

### 2.2. In Vitro miRNA Transfection and Inflammasome Activation

According to the experimental conditions, BMDM cells were seeded in 12-well plates at a density of 1 × 10^6^ cells/well for RNA and protein isolation, 2 × 10^5^ cells/well in Lab-Tek Chamber Slides for immunofluorescence assays, and 5 × 10^5^ cells/well in 96-well plates for Inflammasome-related caspase-1 activity and cell viability. On the following day, cells were transfected with miR-369-3p mimic at two concentrations, 30 and 50 nM (Life Technologies, Carlsbad, CA, USA), using TKO transfection reagent (Mirus Bio LLC, Madison, WI, USA), according to the manufacturer’s instructions. In all experiments, a mock control corresponding to cells transfected with TKO transfection reagent alone was used (Mirus Bio LLC, Madison, WI, USA).

Twenty-four and forty-eight hours after transfection (for RNA and protein extraction respectively), BMDMs were stimulated with Lipopolysaccharide (LPS, Sigma-Aldrich, St. Louis, MO, USA) at a final concentration of 1 μg/mL for 4 h, followed by the addition of Nigericin sodium salt (Sigma-Aldrich, St. Louis, MO, USA) at a final concentration of 20 µM for 30 min.

### 2.3. RNA Isolation and Quantitative Real-Time RT-PCR (qPCR)

Total RNA was extracted using TRIzol reagent (Invitrogen, Carlsbad, CA, USA) according to the manufacturer’s protocol. The RNA concentration was determined using the NanoDrop Spectrophotometer (Thermo Fisher Scientific, Waltham, MA, USA). cDNAs were prepared from 1 μg of total RNAs using iScript Reverse Transcription Supermix (BioRad Laboratories, CA, USA) based on the manufacturer’s recommendations.

qPCR amplification reactions were elicited on a CFX96 System (Biorad Laboratories, Hercules, CA, USA) using SsoAdvanced Universal SYBR Green Supermix (BioRad Laboratories, Hercules, CA, USA) and the primers PrimePCR SYBR green Assay for Brcc3 (BioRad Laboratories, Hercules, CA, USA) and QuantiTect Primer Assay for Nlrp3 and Gapdh (Qiagen, Hilden, Germany). Comparative real-time PCR was performed in triplicate. Gapdh gene amplification was used to normalize the relative expression of the Brcc3 and Nlrp3 genes.

### 2.4. Western Blot Analysis

For total protein extraction, cells were lysed with T-PER Tissue Protein Extraction Reagent (Thermo Fisher Scientific, Waltham, MA, USA) supplemented with a proteinase inhibitors cocktail (Sigma-Aldrich, St. Louis, MO, USA), and the concentration was determined using the Bradford colorimetric assay (Bio-Rad Laboratories, Richmond, CA, USA).

For each sample, 30 µg of proteins were heat-denatured at 100 °C for 5 min in reducing Laemmli Sample Buffer (Biorad Laboratories, Hercules, CA, USA). Proteins were separated on 4–20% Mini-PROTEAN TGX Stain-Free Gels (Biorad Laboratories, Hercules, CA, USA) and transferred onto 0.2 μm pore size PVDF membranes (Biorad Laboratories, Hercules, CA, USA). For protein detection, PVDF membranes were incubated with primary and secondary antibodies in iBind automated Western Systems (Thermo Fisher Scientific, Waltham, MA, USA) following the manufacturer’s instructions. Images were detected using a Chemidoc System (Biorad Laboratories, Hercules, CA, USA), analyzed with Image Lab Software version 5.2.1 (Biorad Laboratories, Hercules, CA, USA), and quantified by ImageJ Software version 1.54d.

Primary antibodies used were rabbit anti-Nlrp3 (#15101, Cell Signaling, Technology, Danvers, MA, USA, dilution 1:1000), rabbit anti-Brcc36 (#18215, Cell Signaling, Technology, Danvers, MA, USA, dilution 1:1000), rabbit anti-Caspase-1 (#24232, Cell Signaling, Technology, Danvers, MA, USA, dilution 1:1000), mouse anti-Gapdh (sc-365062, Santa Cruz Biotechnology, Inc., Heidelberg, Germany, dilution 1:2000). Secondary antibodies used include Goat Anti-mouse IgG-(H+L)-HRP conjugate (170-6516, Biorad Laboratories, Hercules, CA, USA, dilution 1:500) and Goat Anti-rabbit IgG-(H+L)-HRP conjugate (31466, Invitrogen, Carlsbad, CA, USA, dilution 1:2500). The Gapdh protein expression was used to normalize the target protein signal.

### 2.5. Immunoprecipitation

The ubiquitination status of NLRP3 protein was detected using immunoprecipitation. BMDMs were treated with 20 µM of MG132 (Selleckchem, Houston, TX, USA) before LPS and Nigericin stimulations. The pellets were collected and lysed with T-PER Tissue Protein Extraction Reagent (Thermo Fisher Scientific, Waltham, MA, USA) supplemented with proteinase inhibitor cocktail (Sigma-Aldrich, St. Louis, MO, USA). The total protein concentration was established using the Bradford colorimetric assay (Bio-Rad Laboratories, Richmond, CA, USA). Endogenous NLRP3 was immunoprecipitated with an antibody for NLRP3 (#15101, Cell Signaling, Technology, Danvers, MA, USA, 1:50) and Dynabeads Protein A (Thermo Fisher Scientific, Waltham, MA, USA) at room temperature. For each sample, an equal protein concentration was added to the antibody-coated beads and incubated at room temperature. After that, the beads were washed twice with washing buffer, resuspended in Laemmli Sample Buffer 2X (Biorad Laboratories, Hercules, CA, USA), and boiled at 95 °C for 5 min. Immunoprecipitated samples were analyzed by immunoblotting.

### 2.6. ELISA

After transfection and stimulation, cell culture supernatants were collected. The amounts of IL-1β and IL-18 released into the medium were measured by ELISA using mouse IL-1β (R&D Systems, Minneapolis, MN, USA) and mouse IL-18 (R&D Systems, Minneapolis, MN, USA) kits in accordance with the manufacturer’s instructions. Briefly, high-adsorption polystyrene 96-microwell plates (Costar Corning, Corning, NY, USA) were coated overnight with specific capture antibodies in PBS. After washes with PBS + 0.05% Tween-20 (PBST), plates were blocked with 1% BSA in PBS for 1 h at room temperature. Samples diluted in blocking buffer and standard were added to each well and then incubated for 2 h at room temperature. After washes, the captured cytokine was then detected with detection antibody for 2 h. Plates were washed, and the binding was measured after addition of streptavidin–horseradish peroxidase conjugate. and then the reaction was developed with the TMB. The color reaction was stopped with 2N H_2_SO_4_, and the optical density was detected at 450 nm using SPECTROstar Omega Spectrometer (BMG Labtech, Ortenberg, Germany).

### 2.7. Immunofluorescence

Following transfection and stimulation, cells were washed twice with PBS and fixed at room temperature with 4% paraformaldehyde (PFA, Sigma-Aldrich, St. Louis, MO, USA) for 15 min. After washing with PBS, cells were permeabilized and blocked in PBS + BSA 3% and Triton-X 0.1% for 1 h at room temperature. Samples were stained with primary antibody mouse anti-ASC (#67824, Cell Signaling, Technology, Danvers, MA, USA, dilution 1:400) for 3 h at room temperature. Then, cells were incubated with secondary antibody chicken anti-Mouse IgG (H+L) Alexa Fluor 489 (A21200, Invitrogen, Carlsbad, CA, USA, dilution 1:400) in PBS + BSA 3% for 1 h. ProLong Gold Antifade Mountant with DAPI (Thermo Fisher Scientific, Waltham, MA, USA) was applied to each sample, then covered with a glass cover slip. Images were obtained using a fluorescence microscope (Eclipse Ti2, Nikon Inc., Melville, NY, USA). For each sample, three images were captured in different positions.

### 2.8. Measurement of Inflammasome-Related Caspase-1 Activity and Cell Viability

For each sample, an equal volume of supernatant was transferred into 96-well plates and equilibrated at room temperature for 5 min. To detect inflammasome-related caspase-1 activity, an equal volume of the Caspase-Glo Inflammasome Reagent (Promega, Madison, WI, USA) was added in each well according to the manufacturer’s instructions. The plate was incubated at room temperature for 1 h in the dark and the relative luminescence units (RLU) of assays were determined in the LUMIstar Omega microplate luminometer (BMG Labtech, Ortenberg, Germany) according to the manufacturer’s directions.

For cell viability, cells were incubated with Cell Titer Blue Reagent (Promega, Madison, WI, USA) at 37 °C for 1 h according to the manufacturer’s instruction. The relative fluorescence (560Excitation/590Emission) was detected with the FLUOstar Omega microplate spectrophotometer (BMG Labtech, Ortenberg, Germany) following the manufacturer’s instructions.

### 2.9. Immunohistochemistry

Formalin-fixed and paraffin-embedded tissue blocks were obtained from 25 patients subdivided into two groups: the HC group (healthy controls, *n* = 10), consisting of intestinal surgical resections from patients without ulcerative colitis (UC); and the UC group (*n* = 15), including total or subtotal proctocolectomies from patients with longstanding ulcerative colitis refractory to medical therapies. UC patients and controls were retrospectively enrolled in the National Institute of Gastroenterology “S. de Bellis”, Castellana Grotte, Bari, Italy. Written informed consent was obtained from all participants. The study was carried out according to the principles of the Declaration of Helsinki and was approved by the local Institutional Ethics Review Boards (Istituto Tumori Giovanni Paolo II, Bari, Italy, n° 379/2020 of 16 September 2020).

Sections stained with hematoxylin and eosin were reviewed by a pathologist to confirm the adequacy of the sample and to evaluate the morphologic and/or pathological characteristics of the samples. For IHC detection, 4 µm sections were cut and mounted on Apex Bond IHC slides (Leica Biosystems, Buffalo Grove, IL, USA). IHC staining procedures were performed on the BOND III automated immunostainer (Leica Biosystems, Buffalo Grove, IL, USA), from deparaffinization to counterstaining with hematoxylin. Antigen retrieval was carried out by heat-induced antigen retrieval with BOND Epitope Retrieval Solution 1 (Leica Biosystems, Buffalo Grove, IL, USA), a ready to-use, citrate-based pH 6 preparation, for ASC and BRCC3 and with BOND Epitope Retrieval Solution 2 (Leica Biosystems, Buffalo Grove, IL, USA), a ready-to-use EDTA-based pH 9 preparation for NLRP3. Tissue sections were incubated with anti-NLRP3 (PA5-79740, Invitrogen, Waltham, MA, USA, dilution 1:100), anti-BRCC3 (A302-517A, Bethyl Laboratories, Montgomery, TX, USA, dilution 1:1000), and anti-ASC (MA5-26363, Invitrogen, Waltham, MA, USA, dilution 1:200) primary antibodies for 30 min at room temperature. The Bond Polymer Refine Detection Kit (Leica Biosystems, Buffalo Grove, IL, USA) was used as a visualization and chromogen reagent according to the manufacturer’s instructions. Samples were recorded as negative when the number of stained cells was less than 5%.

The positivity expression of all signals in the immune infiltrate was determined through an inflammatory score calculated as follows: 0, absent; 1, ≤25% of positivity; 2, ≤50% of positivity; 3, ≤75% of positivity; 4, ≥75% of positivity.

### 2.10. Bioinformatic and Statistical Analysis

Putative target genes of miR-369-3p were predicted with the miRmap (https://mirmap.ezlab.org/, accessed on 5 December 2022) algorithm.

Statistical analysis was performed using GraphPad Prism software version 9.0.0. Statistical significance of data resulting from different conditions was examined with two-tailed Student’s *t* test, and all values were expressed as the mean ± SEM. Data derive from at least three independent experiments. Differences among experimental conditions were considered statistically significant at *p* < 0.05.

## 3. Results

### 3.1. BRCC3 as Target of miR-369-3p

**Figure 1:**
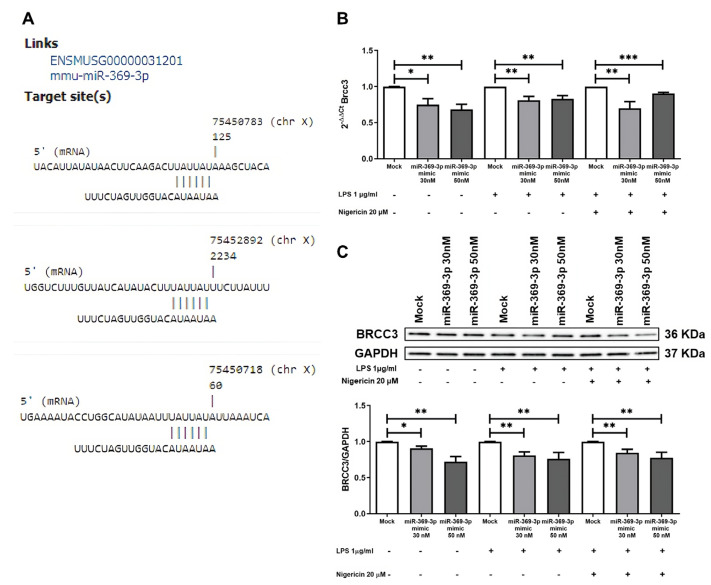
miR-369-3p targets Brrc3. (**A**) Sequence alignments of miR-369-3p and binding sites in 3′ UTR of Brcc3 mRNA. (**B**) The mRNA expression levels of Brcc3 evaluated by qRT-PCR in BMDM cells transfected with 30 nM and 50 nM of miR-369-3p mimic both in the basal condition and after inflammasome activation. (**C**) Western blot analysis of BRCC3 protein expression after miR-369-3p mimic transfection in unstimulated BMDM cells as well as following inflammasome activation of the BMDM cell line. To normalize data, GAPDH was used as housekeeping protein. Data are representative of four independent experiments. Raw data of the independent experiments of Western blot were reported in Supplementary File S1. The histograms correspond to mean ± SEM. (* *p* < 0.05; ** *p* < 0.01; *** *p* < 0.001).

In order to identify new genes targeted by miR-369-3p, we conducted a bioinformatics analysis using the miRmap algorithm (Supplementary Table S1). Among 1717 putative target genes of miR-369-3p, we chose to study Brcc3 for its functional role in regulating NLRP3 inflammasome. In fact, in macrophages, Brcc3 plays a key role in promoting the activation of the inflammasome through deubiquitination of NLRP3 [16]. We found that miR-369-3p links Brcc3 3′UTR in three different sites (Figure 1A).

To functionally demonstrate this target prediction, we conducted studies in the BMDM cell line. Cells were subjected to transient transfection with molecules of miR-369-3p mimic at the concentrations of 30 nM and 50 nM following LPS priming and Nigericin stimulation to activate the NLRP3 inflammasome. Real time-PCR demonstrated that miR-369-3p mimic significantly decreased the Brcc3 mRNA expression level in the basal condition as well as following inflammasome activation (*p* < 0.05; Figure 1B).

Western blot analysis confirmed that miR-369-3p was able to reduce BRCC3 protein expression in unstimulated cells and also after inflammasome-activating stimulations. As shown in Figure 1C, in unstimulated conditions, transient transfection with miR-369-3p mimic results in a reduction in BRCC3 protein levels at both the 30 nM and 50 nM mimic concentrations as compared to mock control (*p* < 0.05; Figure 1C). In addition, after LPS priming and Nigericin stimulation, miR-369-3p mimic decreased the expression level of the BRCC3 protein at both concentrations (*p* < 0.01; Figure 1C).

### 3.2. miR-369-3p Indirectly Regulates the Activation of the NLRP3 Inflammasome

To verify whether the modulation of Brcc3 by miR-369-3p influenced the activation of the inflammasome, we evaluated Nlrp3 expression in BMDM at gene and protein levels.

**Figure 2:**
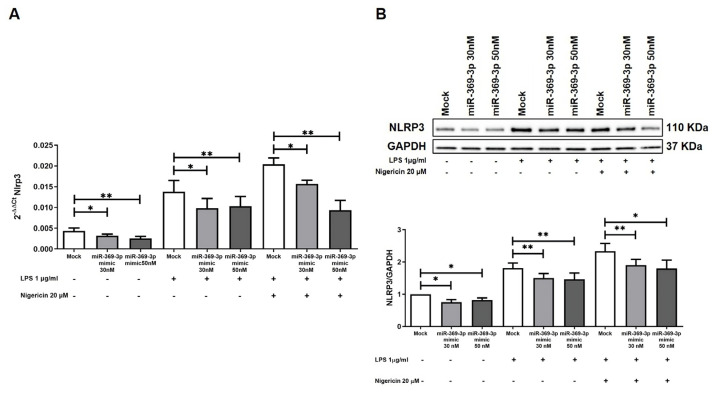
miR-369-3p regulates Nlrp3 mRNA and protein expression. (**A**) The mRNA expression levels of Nlrp3 evaluated by qRT-PCR in BMDM cells transfected with 30 nM and 50 nM of miR-369-3p mimic both in the basal condition and after inflammasome-activating stimulations. (**B**) Western blot analysis of NLRP3 protein expression after miR-369-3p mimic transfection in unstimulated BMDM cells as well as following inflammasome activation in the BMDM cell line. To normalize data, GAPDH was used as housekeeping protein. Data are representative of four independent experiments. Raw data of the independent experiments of Western blot were reported in Supplementary File S1. The histograms correspond to mean ± SEM. (* *p* < 0.05; ** *p* < 0.01).

We investigated the effect of transfection on the expression levels of the Nlrp3 gene after a two-step inflammasomes activation mechanism. Real time-PCR revealed that transient transfection with miR-369-3p mimic at both the 30 nM and 50 nM concentrations significantly decreased Nlrp3 mRNA expression in the basal condition and also in response to LPS priming and Nigericin stimulation (*p* < 0.05; Figure 2A). Moreover, we analyzed the protein expression levels of NLRP3 in BMDM following the transient transfection. As shown by blot images and histograms in Figure 2B, the transient transfection with miR-369-3p mimic decreased the expression levels of NLRP3 at both concentrations in steady-state cells and also following LPS and Nigericin treatment (*p* < 0.05; Figure 2B).

### 3.3. miR-369-3p Modulates the Recruitment of ASC Adaptor Protein

**Figure 3:**
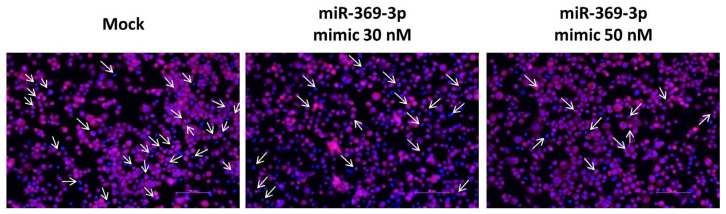
Immunofluorescence staining of ASC in BMDM cell cultures after miR-369-3p mimic transfection. Representative images of BMDM transfected with miR-369-3p mimic and stimulated with LPS 1 μg/mL for 4 h then Nigericin 20 μM for 30 min. Mock condition and transfected conditions were acquired by fluorescence microscopy. Red spots represent the expression of ASC, while DAPI (blue) correspond to cells’ nuclei. White arrows point to ASC expression. Original magnification, ×20. Scale bar presents 50 μm.

Apoptosis-associated speck-like protein containing a CARD (ASC) is an adaptor protein involved in innate immune responses that acts as an essential adapter in the assembly of various inflammasomes [25]. We evaluated the expression of ASC protein by immunofluorescence in BMDM cell cultures after transient transfection of miR-369-3p mimic at 30 nM and 50 nM concentration. In basal condition as well after LPS, the signal of ASC was not detectable. Following stimulation with LPS and Nigericin, ASC expression was detected as spots in the perinuclear portion (Figure 3). The images showed that after transient transfection with the miR-369-3p mimic, ASC expression significantly decreased at both the 30 and 50 nM concentrations compared with the mock control in Nigericin-stimulated conditions (Figure 3).

### 3.4. miR-369-3p Regulates Deubiquitination of NLRP3 Inflammasomes

**Figure 4:**
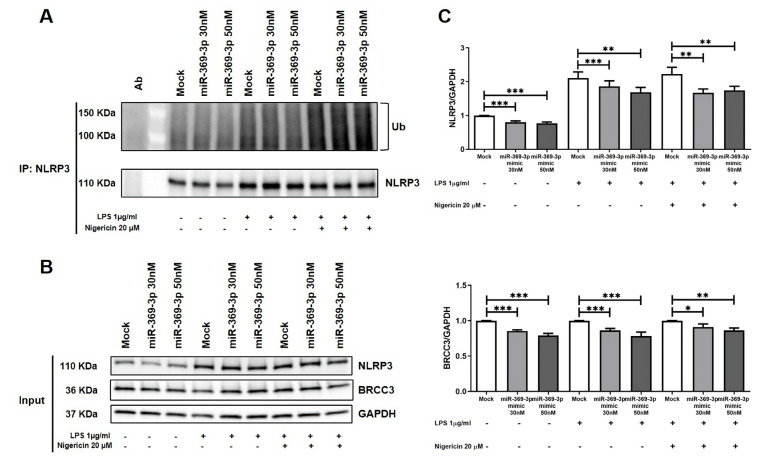
miR-369-3p modulated the deubiquitination of NLRP3 by BRCC3. (**A**) Endogenous NLRP3 immunoprecipitates were analyzed for ubiquitination. miR-369-3p modulated the deubiquitination of NLRP3 by BRCC3 after miR-369-3p transient transfection and LPS and Nigericin stimulation. (**B**) Expression of NLRP3 and BRCC3 in BMDM lysates after miR-369-3p transient transfection and LPS and Nigericin stimulation (input samples). (**C**) Histograms of NLRP3 and BRCC3 expression from lysates of BMDM treated with miR-369-3p mimic and stimulated with LPS and Nigericin. To normalize data, GAPDH was used as housekeeping protein. Raw data of the independent experiments of Western blot were reported in Supplementary File S1. The histograms correspond to mean ± SEM. Data are representative of four independent experiments. (* *p* < 0.05; ** *p* < 0.01; *** *p* < 0.001).

BRCC3 was identified as a regulator of ubiquitination with the consequent activation of NLRP3. Then, we evaluated the ubiquitin levels of NLRP3 after stimulation with LPS and Nigericin through immunoprecipitation. We determined whether the overexpression of miR-369-3p modulated the ubiquitination levels of NLRP3. Immunoprecipitation assays performed in BMDMs stimulated with LPS and Nigericin showed that miR-369-3p mimic diminished the activity of BRCC3, decreasing the deubiquitination of NLRP3 (Figure 4A). Further demonstrating the above results, we found that, also in the input samples, the increase in miR-369-3p reduced the expression of BRCC3 and consequently modulated the expression of NLRP3 after LPS and Nigericin stimulation (Figure 4B,C).

### 3.5. miR-369-3p Reduced the Activation and Release of Caspase-1

Caspase-1 (CASP1) is a protease produced and stored as an inactive zymogen (pro-caspase-1). Activation of CASP1 is an important functional output downstream of the NLRP3 inflammasome, directly involved in the processing and release of mature pro-inflammatory cytokines.

**Figure 5:**
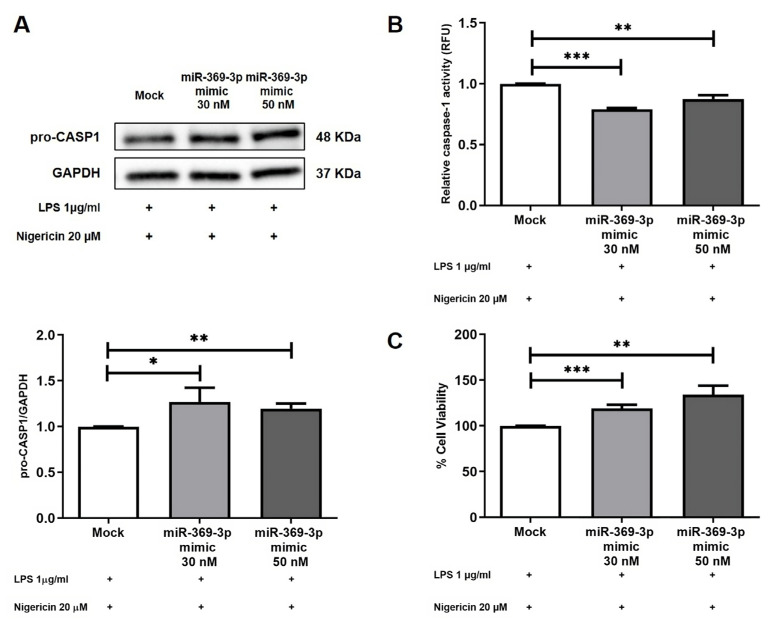
miR-369-3p reduced the activation and release of caspase-1. (**A**) Western blot analysis of pro-CASP1 protein expression after miR-369-3p mimic transfection following LPS and Nigericin stimulations in the BMDM cell line. To normalize data, GAPDH was used as housekeeping protein. Raw data of the independent experiments of Western blot were reported in Supplementary File S1. (**B**) Caspase-1 activity after LPS and Nigericin stimulations in the mimic transfected BMDM cell line. (**C**) In vitro cell viability assay of BMDM cells transiently transfected with miR-369-3p mimic and stimulated with LPS and Nigericin. (* *p* < 0.05; ** *p* < 0.01; *** *p* < 0.0001).

To evaluate the expression levels of CASP1 in BMDM cell cultures, we firstly analyzed the expression levels of pro-CASP1 protein after transient transfection and LPS and Nigericin stimulation. We found that miR-369-3p mimic was able to significantly increase its expression at both the 30 nM and 50 nM concentrations compared to mock control (*p* < 0.05; Figure 5A). To confirm the reduction in NLRP3 inflammasome activation, we examined the caspase-1 activity. As shown in the histogram in Figure 5B, the activity resulted in being significantly reduced at both mimic concentrations compared to the mock control (*p* < 0.001; Figure 5B).

In some instances, caspase-1 activation by inflammasomes may trigger pyroptosis, a highly inflammatory form of programmed cell death [26]. Thus, we assessed BMDM cell viability subsequent to the miR-369-3p mimic transfection. We demonstrated that miR-369-3p determined an increased cell viability compared to the mock control (*p* < 0.001; Figure 5C).

### 3.6. Effect of miR-369-3p on Release of Inflammasome-Related Pro-Inflammatory Cytokines

**Figure 6:**
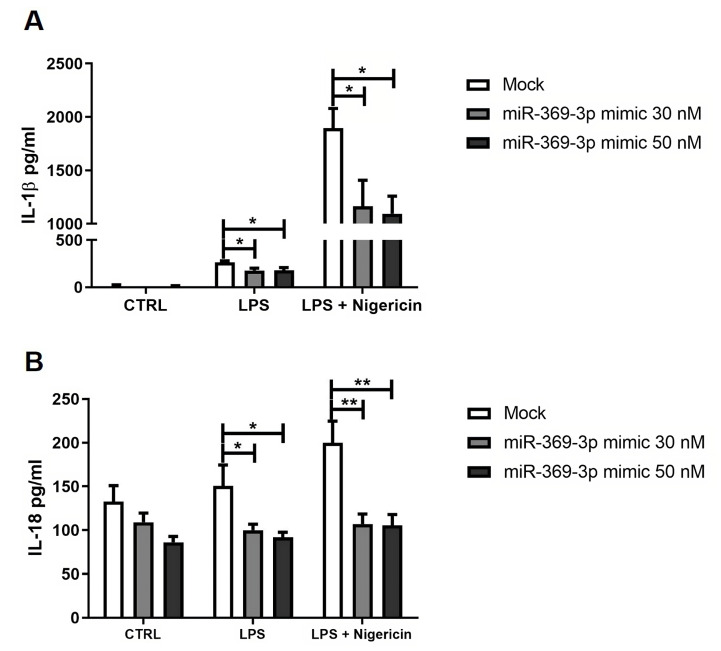
miR-369-3p modulated the release of pro-inflammatory cytokines, NLRP3 inflammasome-related. miR-369-3p induction in BMDM after mimic transfection led to a significant decrease in IL-1β (**A**), IL-18 (**B**) production in response to LPS and LPS and Nigericin stimulation. Data are representative of four independent experiments. (* *p* < 0.05; ** *p* < 0.01).

To investigate whether the expression of miR-369-3p was functionally involved in the production of cytokines by inflammasomes, we transfected BMDM with miR-369-3p mimic molecules, and then analyzed the levels of IL-1β and IL-18 cytokines in the cell-free supernatants after LPS and Nigericin stimulation. In relation to cells without stimulation, the LPS led to an increase in IL-1β and IL-18 cytokines. However, miR-369-3p induction significantly decreased the production of IL-1β (*p* < 0.05; Figure 6A) and IL-18 (*p* < 0.05; Figure 6B) cytokines in response to LPS. The activation of caspase-1 by inflammasomes is responsible for proteolytic scission of pro-IL-1β and pro-IL-18. For this reason, we evaluated the expression levels of pro-inflammatory cytokines related to caspase-1 activation, analysing cell-free supernatants of BMDMs transfected with miR-369-3p after combined LPS and Nigericin stimulation. Also in this case, the gain of miR-369-3p significantly decreased IL-1β (*p* < 0.05; Figure 6A) and IL-18 (*p* < 0.01; Figure 6B) at both miRNA concentrations compared to the mock control.

### 3.7. BRCC3, NLRP3, and ASC Expression in UC Patients

**Figure 7:**
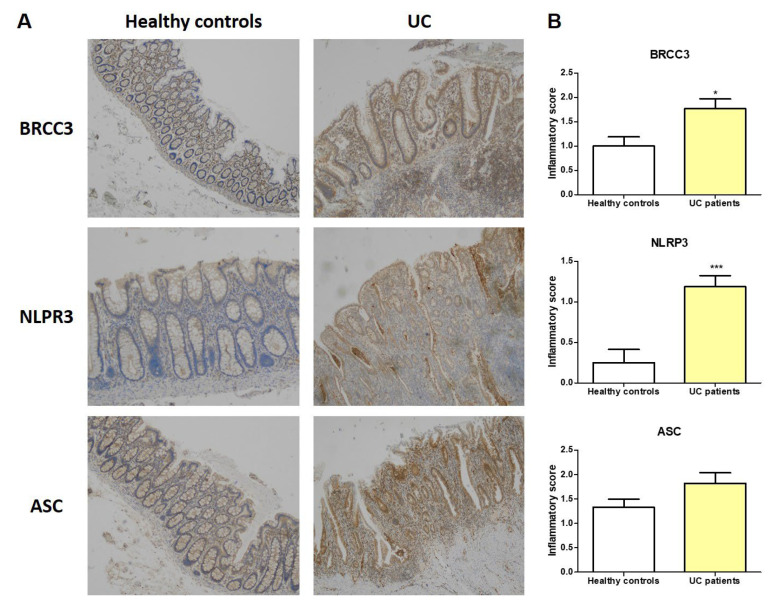
BRCC3 and NLRP3 and ASC expression in UC patients. (**A**) BRCC3, NLRP3, and ASC protein expression in formalin-fixed, paraffin-embedded tissues obtained from healthy controls and ulcerative colitis (UC) patients. Original magnification, ×4. (**B**) Inflammatory score representing the expression levels of BRCC3, NLRP3, and ASC proteins in the immune infiltrate (* *p* < 0.01; *** *p* < 0.001).

We evaluated the different expression of BRCC3, NLRP3, and ASC proteins on tissue specimens of UC patients and healthy controls collected at our Institute. IHC showed that BRCC3, NLRP3, and ASC expression levels were higher in the UC tissues compared to healthy control tissues (Figure 7A). Moreover, we found that in tissues of UC patients, these three proteins showed an intense and diffuse positivity in the immune infiltrate (Figure 7A). We also defined the grade of positive immunostaining by determining the inflammatory score. We found that NLRP3 and BRCC3 were significantly higher in UC patients than healthy controls (*p* < 0.01; Figure 7B); instead, ASC showed an increase, but this did not reach statistical significance (*p* > 0.05; Figure 7B). Notably, these three proteins were also expressed in epithelial cells, and their expression was increased in UC tissues compared to healthy controls (Figure 7A), confirming previous data on NLRP3 inflammasome expression in gut epithelial cells [5,27].

## 4. Discussion

The inflammasomes are multiprotein complexes located in myeloid cells, principally in macrophages, that act as receptors and sensors of the immune system to detect various endogenous and exogenous stimuli and to induce immune responses. The assembly of the inflammasome is a critical and well-organized process, and is fundamental for the host defences in response to foreign pathogens or tissue damage [28]. Aberrant inflammasome activation leads to uncontrolled responses by immune cells that may contribute to various diseases and inflammatory disorders [29,30,31].

Activation of NLRP3 is considered an important step in the pathogenesis of IBD since it is involved in the inflammatory process affecting intestinal epithelial cells and macrophages [6]. In non-immune cells such as human umbilical cord blood-derived mesenchymal stem cells (hUCB-MSCs), in vitro as well as in DSS-induced colitis, NLRP3 inflammasome activation improved the protective anti-inflammatory abilities of several immune cells, including macrophages [32]. However, the role of the NLRP3 inflammasome in acute intestinal inflammation is controversial, as contradictory studies have identified opposing functions during experimental colitis. Several studies demonstrated that the loss of inflammasome functions led to a reduction in many mechanisms associated with the IBD inflammatory state [6,11,33]. Moreover, mice deficient in inflammasome components such as NLRP3, caspase-1, or ASC have an enhanced susceptibility to DSS-colitis [34,35,36]. On the contrary, other studies in animal models supported the protective effects of gut inflammasomes in the development of IBD [37]. These discrepancies across studies may be explained by the experimental design and the opposing role of IL-1β versus IL-18 that can drive both inflammation and repair [38,39]. Furthermore, recurrent intestinal tissue injury and prolonged inflammation can transform the protective role of inflammasomes into a harmful effect [6].

The involvement of miRNAs in several signaling pathways that are altered in IBD demonstrated their potential as a targeted therapy in the treatment of these patients. Some studies have reported that some miRNAs were associated with several physio-pathological features of IBD. miRNAs can regulate the inflammatory responses by immune cells and the barrier function of epithelial cells [40,41,42,43]. In our previous studies, we also demonstrated that miR-369-3p acted by regulating the expression levels of NOS2 in LPS-induced inflammatory responses, thus decreasing the production of pro-inflammatory agents in dendritic cells [22,23]. In addition, we showed that miR-369-3p modulated the expression and activities of immunoproteasomes through regulation of the PSMB9 subunit [24]. Moreover, we functionally characterized, in intestinal epithelial cells, the role of miR-195-5p in the regulation of TJ protein expression and barrier integrity, and demonstrated that miR-195-5p modulated the expression of CLDN2 and the other TJ protein expression, also under inflammatory conditions [44,45].

In the last few years, research into NLRP3 regulation by miRNAs has demonstrated great potential in the treatment of several diseases, including inflammatory diseases, autoimmune disorders, and cancers [46]. NLRP3 inflammasome activation is closely associated with Parkinson’s disease pathogenesis. Zhou and colleagues demonstrated that enhancing miR-7 expression levels significantly reduced NLRP3 levels, ameliorating neuroinflammation [47]. Pan and co-workers reported that miR-23a regulated the neuropathic pain by targeting CXCR4 and suppressed NLRP3 inflammation activation via the TXNIP/NLRP3 inflammasome axis [48]. miR-223 was demonstrated to be an important targeted therapy for IBD since it was identified as a critical regulator of the NLRP3 inflammasome. Indeed, miR-223 suppressed NLRP3 expression, reduced IL-1β production, and thus ameliorated the experimental colitis [49].

In the present study, we demonstrated that miR-369-3p was able to modulate the expression of the BRCC3 and NLRP3 inflammasomes. The formation of the NLRP3 inflammasome is closely regulated by a two-step process consisting of priming and activation signals [10]. Upon activation, NLRP3 oligomerized and recruited ASC and pro-caspase-1 to form the NLRP3 inflammasome complex. This assembly triggered the cleavage of pro-caspase-1 and the subsequent maturation of pro-inflammatory cytokines IL-1β and IL-18. In addition, NLRP3 activation may require combinations of different post-translational modifications such as deubiquitination. BRCC3 is a deubiquitinating enzyme that plays an essential role for the efficient assembly and activation of NLRP3 inflammasome. BRCC3 binds directly to NLRP3 and promotes the NLRP3 inflammasome activation through its deubiquitination [13].

Here, we report that transfection with miR-369-3p mimic was able to reduce the expression of NLRP3 via BRCC3 modulation following inflammasome activation. The increase in miR-369-3p was able to significantly reduce the expression of the BRCC3 gene and protein. This modulation, in turn, influenced the recruitment of NLRP3 and thus the formation of the inflammasome complex. We also demonstrated that miR-369-3p indirectly blocks the recruitment of ASC adaptor protein by NLRP3. In addition, after raising the intracellular levels of miR-369-3p, Caspase-1 activation by inflammasomes was reduced, decreasing the cleavage of pro-IL-1β and pro-IL-18.

In UC patients, we also confirmed that BRCC3 as well as NLRP3 and ASC were increased as compared to in healthy controls, mostly in the immune infiltrate. These three proteins were also expressed in the epithelium, with an increased expression in UC tissue, confirming other previous studies [5,27]. The epithelium contributes to the recognition and transmission of inflammatory signals, acting as an initial sensor of danger and executor of the response. In this context, inflammasome activation in the epithelium was associated with severe intestinal inflammation [50]. We supposed that miR-369-3p could also act in intestinal epithelial cells by regulating the activity of inflammasomes, but further studies are required to prove this regulation.

To date, several agents that targeted the inflammasome pathways have been discovered. These small molecule inhibitors are designed to influence NLRP3 expression, degradation, and manipulation of NLRP3 function. MCC950 is considered one of the most potent and selective pharmacological inhibitors of the NLRP3 inflammasome. In mouse colitis model, MCC950 was demonstrated to act on NLRP3 by inhibiting ASC oligomerization and caspase-1 dependent activation of IL-1β and IL-18, and reducing the levels of pro-inflammatory cytokines [51]. Oridonin, a bioactive diterpenoid, is another covalent inhibitor of NLRP3 that was shown to suppress experimental colitis in mice [52]. Other molecules target ASC, PAMPs, and DAMPs [6]. Sulforaphane (a potential caspase-1 inhibitor) and Anakinra (IL-1β receptor antagonist) are other molecules that were explored for the treatment of IBD [53,54]. However, each molecule is able to regulate a single step in the inflammasome process. Here, we propose a novel mechanism that, by acting on post-translational modification of NLRP3 inflammasome activation, may be an interesting new target in personalized treatment of inflammatory disorders, including IBD.

## 5. Conclusions

In conclusion, our results support the therapeutic potential that miR-369-3p may have, by acting on the expression of the NLRP3 inflammasome via BRCC3. We demonstrate that miR-369-3p may ameliorate the inflammatory state of IBD patients by modulating the NLRP3 inflammasome complex, thereby suggesting potential new therapeutic approaches in intestinal inflammation.

## 6. Patents

An Italian patent entitled “Pharmaceutical composition based on miR-369-3p as ac-tive ingredient for the treatment of chronic inflammatory disorders” (patent n° 102018000007954) was issued on 3 August 2020 to the Ente Ospedaliero Specializzato in Gastroenterologia “Saverio de Bellis”.

## Data Availability

Not applicable.

## Supplementary Materials

The following supporting information can be downloaded at: https://www.mdpi.com/article/10.3390/cells12172184/s1, Table S1: List of miR-369-3p target genes; File S1: Raw data of the independent experiments of Western blot.
