# Supplementary material for: miR-369-3p Modulates Intestinal Inflammatory Response via BRCC3/NLRP3 Inflammasome Axis
Source: Cells. 2023 Aug 31;12(17):2184. doi: 10.3390/cells12172184 (PMC10486421; doi:10.3390/cells12172184)
Supplement: Supplementary file 1 [file cells-12-02184-s001.zip › File S1.pdf]

Western blot for BRCC3

Experiment 1

BRCC3

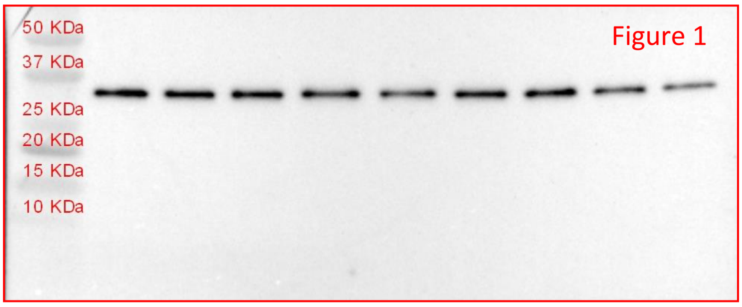

Lane 1 Standard Molecular Weight
Lane 2 Mock
Lane 3 miR-369-3p mimic 30 nM
Lane 4 miR-369-3p mimic 50 nM
Lane 5 Mock + LPS 1 µg/ml 4h
Lane 6 miR-369-3p mimic 30 nM + LPS 1 µg/ml 4h
Lane 7 miR-369-3p mimic 50 nM + LPS 1 µg/ml 4h
Lane 8 Mock + LPS 1 µg/ml 4h + Nigericin 20 µM 30 min
Lane 9 miR-369-3p mimic 30 nM + LPS 1 µg/ml 4h + Nigericin 20 µM 30 min
Lane 10 miR-369-3p mimic 50 nM + LPS 1 µg/ml 4h + Nigericin 20 µM 30 min

GAPDH

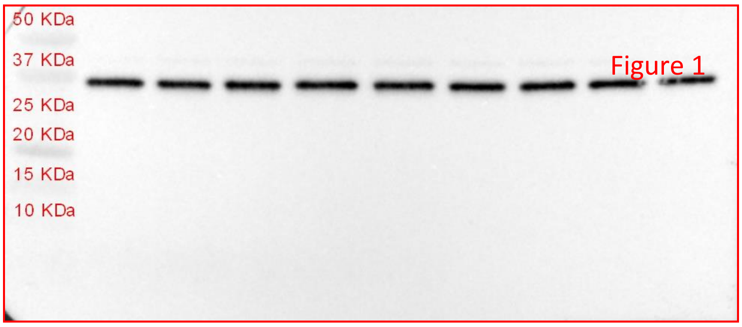

Lane 1 Standard Molecular Weight
Lane 2 Mock
Lane 3 miR-369-3p mimic 30 nM
Lane 4 miR-369-3p mimic 50 nM
Lane 5 Mock + LPS 1 µg/ml 4h
Lane 6 miR-369-3p mimic 30 nM + LPS 1 µg/ml 4h
Lane 7 miR-369-3p mimic 50 nM + LPS 1 µg/ml 4h
Lane 8 Mock + LPS 1 µg/ml 4h + Nigericin 20 µM 30 min
Lane 9 miR-369-3p mimic 30 nM + LPS 1 µg/ml 4h + Nigericin 20 µM 30 min
Lane 10 miR-369-3p mimic 50 nM + LPS 1 µg/ml 4h + Nigericin 20 µM 30 min

Experiment 2

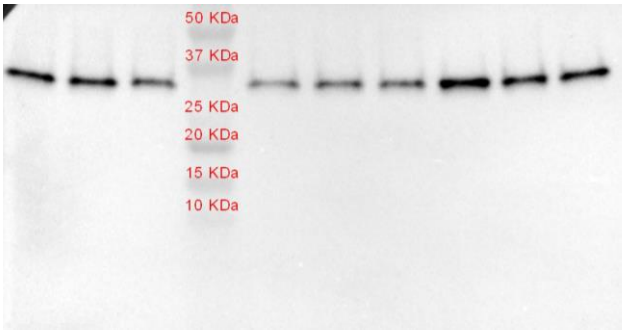

BRCC3

Lane 1 Mock
Lane 2 miR-369-3p mimic 30 nM
Lane 3 miR-369-3p mimic 50 nM
Lane 4 Standard Molecular Weight
Lane 5 Mock + LPS 1 µg/ml 4h
Lane 6 miR-369-3p mimic 30 nM + LPS 1 µg/ml 4h
Lane 7 miR-369-3p mimic 50 nM + LPS 1 µg/ml 4h
Lane 8 Mock + LPS 1 µg/ml 4h + Nigericin 20 µM 30 min
Lane 9 miR-369-3p mimic 30 nM + LPS 1 µg/ml 4h + Nigericin 20 µM 30 min
Lane 10 miR-369-3p mimic 50 nM + LPS 1 µg/ml 4h + Nigericin 20 µM 30 min

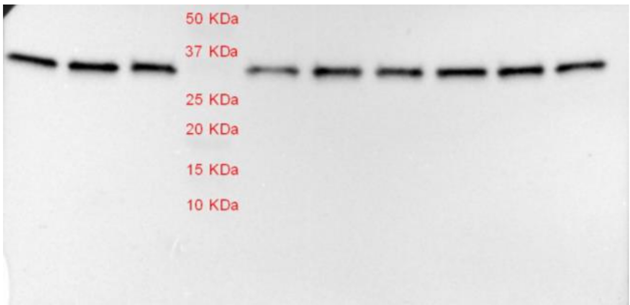

GAPDH

Lane 1 Mock
Lane 2 miR-369-3p mimic 30 nM
Lane 3 miR-369-3p mimic 50 nM
Lane 4 Standard Molecular Weight
Lane 5 Mock + LPS 1 µg/ml 4h
Lane 6 miR-369-3p mimic 30 nM + LPS 1 µg/ml 4h
Lane 7 miR-369-3p mimic 50 nM + LPS 1 µg/ml 4h
Lane 8 Mock + LPS 1 µg/ml 4h + Nigericin 20 µM 30 min
Lane 9 miR-369-3p mimic 30 nM + LPS 1 µg/ml 4h + Nigericin 20 µM 30 min
Lane 10 miR-369-3p mimic 50 nM + LPS 1 µg/ml 4h + Nigericin 20 µM 30 min

Experiment 3

BRCC3

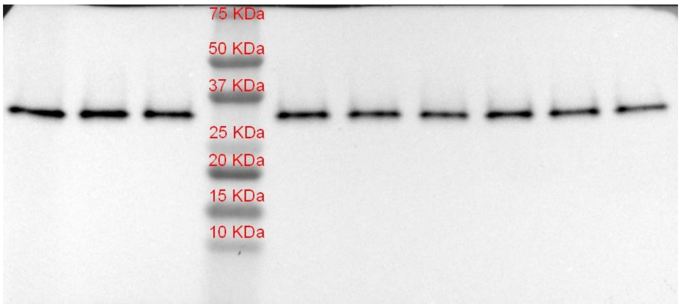

Lane 1 Mock
Lane 2 miR-369-3p mimic 30 nM
Lane 3 miR-369-3p mimic 50 nM
Lane 4 Standard Molecular Weight
Lane 5 Mock + LPS 1 µg/ml 4h
Lane 6 miR-369-3p mimic 30 nM + LPS 1 µg/ml 4h
Lane 7 miR-369-3p mimic 50 nM + LPS 1 µg/ml 4h
Lane 8 Mock + LPS 1 µg/ml 4h + Nigericin 20 µM 30 min
Lane 9 miR-369-3p mimic 30 nM + LPS 1 µg/ml 4h + Nigericin 20 µM 30 min
Lane 10 miR-369-3p mimic 50 nM + LPS 1 µg/ml 4h + Nigericin 20 µM 30 min

GAPDH

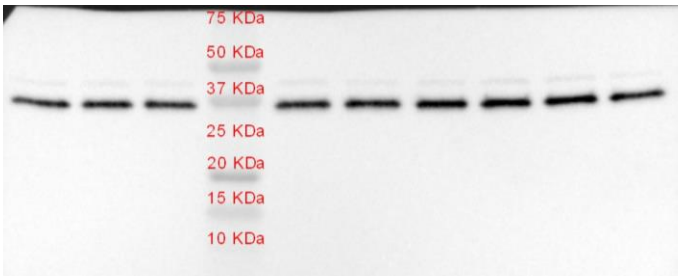

Lane 1 Mock
Lane 2 miR-369-3p mimic 30 nM
Lane 3 miR-369-3p mimic 50 nM
Lane 4 Standard Molecular Weight
Lane 5 Mock + LPS 1 µg/ml 4h
Lane 6 miR-369-3p mimic 30 nM + LPS 1 µg/ml 4h
Lane 7 miR-369-3p mimic 50 nM + LPS 1 µg/ml 4h
Lane 8 Mock + LPS 1 µg/ml 4h + Nigericin 20 µM 30 min
Lane 9 miR-369-3p mimic 30 nM + LPS 1 µg/ml 4h + Nigericin 20 µM 30 min
Lane 10 miR-369-3p mimic 50 nM + LPS 1 µg/ml 4h + Nigericin 20 µM 30 min

Experiment 4

BRCC3

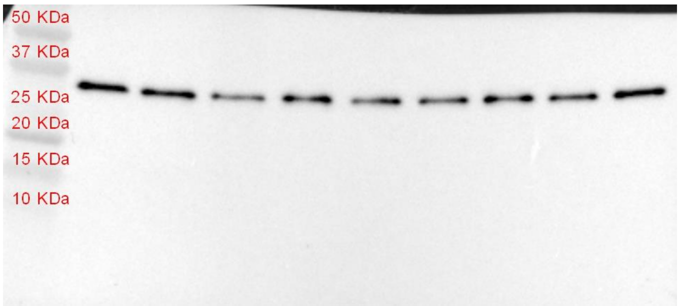

Lane 1 Standard Molecular Weight
Lane 2 Mock
Lane 3 miR-369-3p mimic 30 nM
Lane 4 miR-369-3p mimic 50 nM
Lane 5 Mock + LPS 1 µg/ml 4h
Lane 6 miR-369-3p mimic 30 nM + LPS 1 µg/ml 4h
Lane 7 miR-369-3p mimic 50 nM + LPS 1 µg/ml 4h
Lane 8 Mock + LPS 1 µg/ml 4h + Nigericin 20 µM 30 min
Lane 9 miR-369-3p mimic 30 nM + LPS 1 µg/ml 4h + Nigericin 20 µM 30 min
Lane 10 miR-369-3p mimic 50 nM + LPS 1 µg/ml 4h + Nigericin 20 µM 30 min

GAPDH

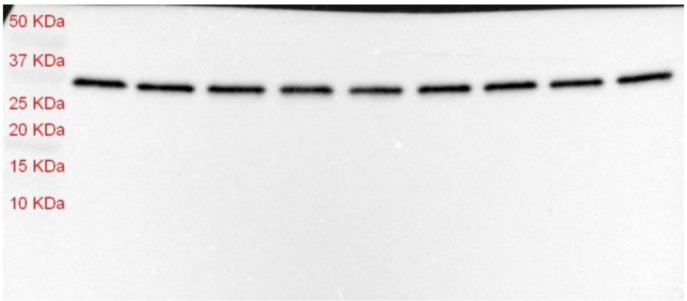

Lane 1 Standard Molecular Weight
Lane 2 Mock
Lane 3 miR-369-3p mimic 30 nM
Lane 4 miR-369-3p mimic 50 nM
Lane 5 Mock + LPS 1 µg/ml 4h
Lane 6 miR-369-3p mimic 30 nM + LPS 1 µg/ml 4h
Lane 7 miR-369-3p mimic 50 nM + LPS 1 µg/ml 4h
Lane 8 Mock + LPS 1 µg/ml 4h + Nigericin 20 µM 30 min
Lane 9 miR-369-3p mimic 30 nM + LPS 1 µg/ml 4h + Nigericin 20 µM 30 min
Lane 10 miR-369-3p mimic 50 nM + LPS 1 µg/ml 4h + Nigericin 20 µM 30 min

Western blot for NLRP3

Experiment 1

NLRP3

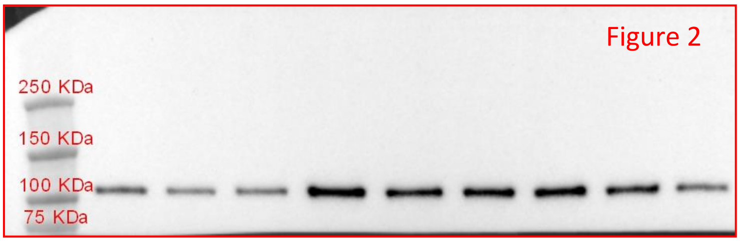

Lane 1 Standard Molecular Weight
Lane 2 Mock
Lane 3 miR-369-3p mimic 30 nM
Lane 4 miR-369-3p mimic 50 nM
Lane 5 Mock + LPS 1 µg/ml 4h
Lane 6 miR-369-3p mimic 30 nM + LPS 1 µg/ml 4h
Lane 7 miR-369-3p mimic 50 nM + LPS 1 µg/ml 4h
Lane 8 Mock + LPS 1 µg/ml 4h + Nigericin 20 µM 30 min
Lane 9 miR-369-3p mimic 30 nM + LPS 1 µg/ml 4h + Nigericin 20 µM 30 min
Lane 10 miR-369-3p mimic 50 nM + LPS 1 µg/ml 4h + Nigericin 20 µM 30 min

GAPDH

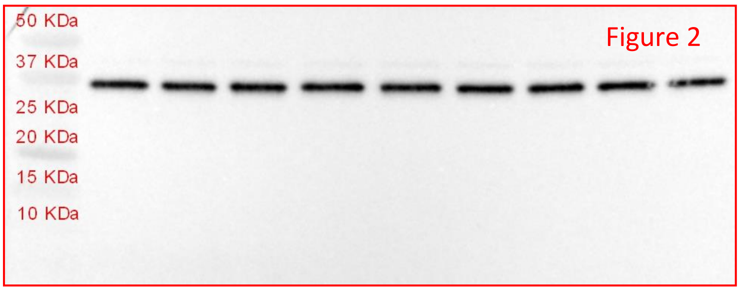

Lane 1 Standard Molecular Weight
Lane 2 Mock
Lane 3 miR-369-3p mimic 30 nM
Lane 4 miR-369-3p mimic 50 nM
Lane 5 Mock + LPS 1 µg/ml 4h
Lane 6 miR-369-3p mimic 30 nM + LPS 1 µg/ml 4h
Lane 7 miR-369-3p mimic 50 nM + LPS 1 µg/ml 4h
Lane 8 Mock + LPS 1 µg/ml 4h + Nigericin 20 µM 30 min
Lane 9 miR-369-3p mimic 30 nM + LPS 1 µg/ml 4h + Nigericin 20 µM 30 min
Lane 10 miR-369-3p mimic 50 nM + LPS 1 µg/ml 4h + Nigericin 20 µM 30 min

Experiment 2

NLRP3

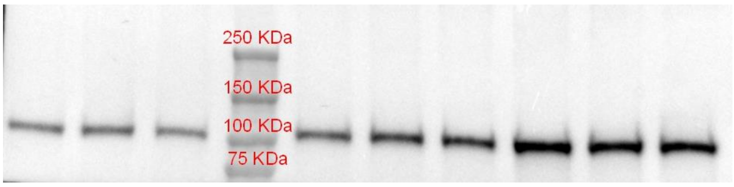

Lane 1 Mock
Lane 2 miR-369-3p mimic 30 nM
Lane 3 miR-369-3p mimic 50 nM
Lane 4 Standard Molecular Weight
Lane 5 Mock + LPS 1 µg/ml 4h
Lane 6 miR-369-3p mimic 30 nM + LPS 1 µg/ml 4h
Lane 7 miR-369-3p mimic 50 nM + LPS 1 µg/ml 4h
Lane 8 Mock + LPS 1µg/ml 4h + Nigericin 20µM 30 min
Lane 9 miR-369-3p mimic 30 nM + LPS 1 µg/ml 4h + Nigericin 20 µM 30 min
Lane 10 miR-369-3p mimic 50 nM + LPS 1 µg/ml 4h + Nigericin 20 µM 30 min

GAPDH

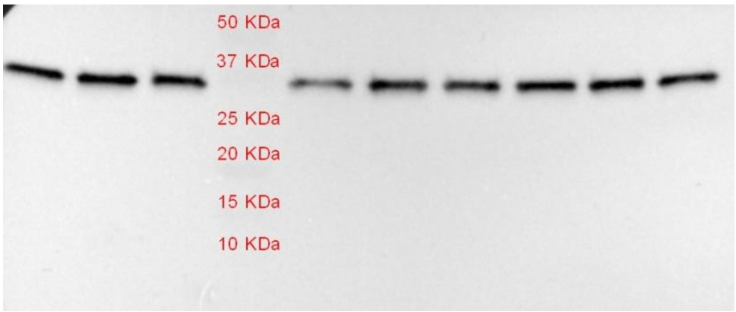

Lane 1 Mock
Lane 2 miR-369-3p mimic 30 nM
Lane 3 miR-369-3p mimic 50 nM
Lane 4 Standard Molecular Weight
Lane 5 Mock + LPS 1 µg/ml 4h
Lane 6 miR-369-3p mimic 30 nM + LPS 1 µg/ml 4h
Lane 7 miR-369-3p mimic 50 nM + LPS 1 µg/ml 4h
Lane 8 Mock + LPS 1µg/ml 4h + Nigericin 20µM 30 min
Lane 9 miR-369-3p mimic 30 nM + LPS 1 µg/ml 4h + Nigericin 20 µM 30 min
Lane 10 miR-369-3p mimic 50 nM + LPS 1 µg/ml 4h + Nigericin 20 µM 30 min

Experiment 3
NLRP3

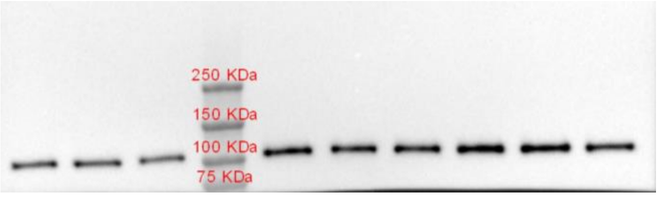

Lane 1 Mock
Lane 2 miR-369-3p mimic 30 nM
Lane 3 miR-369-3p mimic 50 nM
Lane 4 Standard Molecular Weight
Lane 5 Mock + LPS 1 µg/ml 4h
Lane 6 miR-369-3p mimic 30 nM + LPS 1 µg/ml 4h
Lane 7 miR-369-3p mimic 50 nM + LPS 1 µg/ml 4h
Lane 8 Mock + LPS 1µg/ml 4h + Nigericin 20µM 30 min
Lane 9 miR-369-3p mimic 30 nM + LPS 1 µg/ml 4h + Nigericin 20 µM 30 min
Lane 10 miR-369-3p mimic 50 nM + LPS 1 µg/ml 4h + Nigericin 20 µM 30 min

GAPDH

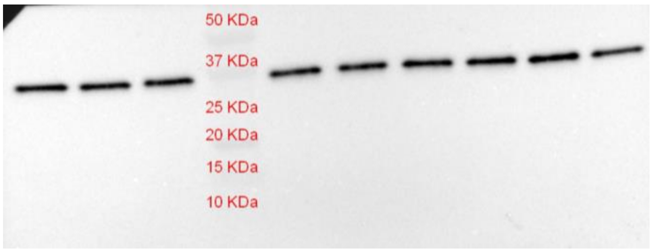

Lane 1 Mock
Lane 2 miR-369-3p mimic 30 nM
Lane 3 miR-369-3p mimic 50 nM
Lane 4 Standard Molecular Weight
Lane 5 Mock + LPS 1 µg/ml 4h
Lane 6 miR-369-3p mimic 30 nM + LPS 1 µg/ml 4h
Lane 7 miR-369-3p mimic 50 nM + LPS 1 µg/ml 4h
Lane 8 Mock + LPS 1µg/ml 4h + Nigericin 20µM 30 min
Lane 9 miR-369-3p mimic 30 nM + LPS 1 µg/ml 4h + Nigericin 20 µM 30 min
Lane 10 miR-369-3p mimic 50 nM + LPS 1 µg/ml 4h + Nigericin 20 µM 30 min

Experiment 4
NLRP3

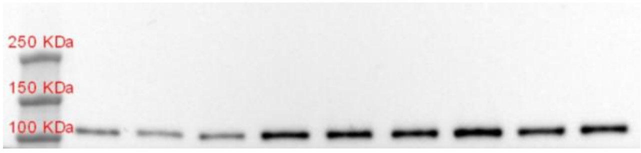

Lane 1 Standard Molecular Weight
Lane 2 Mock
Lane 3 miR-369-3p mimic 30 nM
Lane 4 miR-369-3p mimic 50 nM
Lane 5 Mock + LPS 1 µg/ml 4h
Lane 6 miR-369-3p mimic 30 nM + LPS 1 µg/ml 4h
Lane 7 miR-369-3p mimic 50 nM + LPS 1 µg/ml 4h
Lane 8 Mock + LPS 1 µg/ml 4h + Nigericin 20 µM 30 min
Lane 9 miR-369-3p mimic 30 nM + LPS 1 µg/ml 4h + Nigericin 20 µM 30 min
Lane 10 miR-369-3p mimic 50 nM + LPS 1 µg/ml 4h + Nigericin 20 µM 30 min

GAPDH

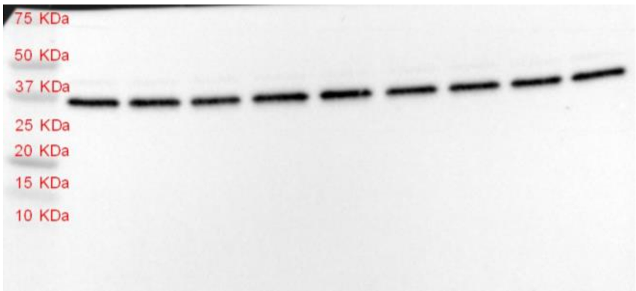

Lane 1 Standard Molecular Weight
Lane 2 Mock
Lane 3 miR-369-3p mimic 30 nM
Lane 4 miR-369-3p mimic 50 nM
Lane 5 Mock + LPS 1 µg/ml 4h
Lane 6 miR-369-3p mimic 30 nM + LPS 1 µg/ml 4h
Lane 7 miR-369-3p mimic 50 nM + LPS 1 µg/ml 4h
Lane 8 Mock + LPS 1 µg/ml 4h + Nigericin 20 µM 30 min
Lane 9 miR-369-3p mimic 30 nM + LPS 1 µg/ml 4h + Nigericin 20 µM 30 min
Lane 10 miR-369-3p mimic 50 nM + LPS 1 µg/ml 4h + Nigericin 20 µM 30 min

Western blot for Caspase-1

Experiment 1

Caspase-1

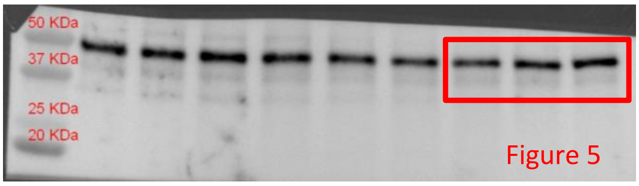

Lane 1 Standard Molecular Weight
Lane 2 Mock
Lane 3 miR-369-3p mimic 30 nM
Lane 4 miR-369-3p mimic 50 nM
Lane 5 Mock + LPS 1 µg/ml 4h
Lane 6 miR-369-3p mimic 30 nM + LPS 1 µg/ml 4h
Lane 7 miR-369-3p mimic 50 nM + LPS 1 µg/ml 4h
Lane 8 Mock + LPS 1 µg/ml 4h + Nigericin 20 µM 30 min
Lane 9 miR-369-3p mimic 30 nM + LPS 1 µg/ml 4h + Nigericin 20 µM 30 min
Lane 10 miR-369-3p mimic 50 nM + LPS 1 µg/ml 4h + Nigericin 20 µM 30 min

GAPDH

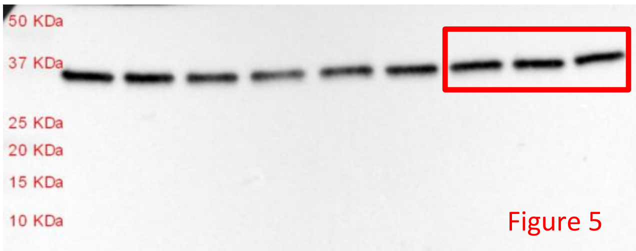

Lane 1 Standard Molecular Weight
Lane 2 Mock
Lane 3 miR-369-3p mimic 30 nM
Lane 4 miR-369-3p mimic 50 nM
Lane 5 Mock + LPS 1 µg/ml 4h
Lane 6 miR-369-3p mimic 30 nM + LPS 1 µg/ml 4h
Lane 7 miR-369-3p mimic 50 nM + LPS 1 µg/ml 4h
Lane 8 Mock + LPS 1 µg/ml 4h + Nigericin 20 µM 30 min
Lane 9 miR-369-3p mimic 30 nM + LPS 1 µg/ml 4h + Nigericin 20 µM 30 min
Lane 10 miR-369-3p mimic 50 nM + LPS 1 µg/ml 4h + Nigericin 20 µM 30 min

Experiment 2

Caspase-1

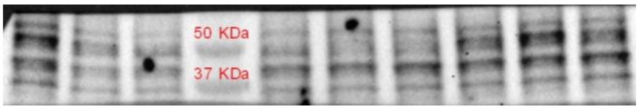

Lane 1 Mock
Lane 2 miR-369-3p mimic 30 nM
Lane 3 miR-369-3p mimic 50 nM
Lane 4 Standard Molecular Weight
Lane 5 Mock + LPS 1 µg/ml 4h
Lane 6 miR-369-3p mimic 30 nM + LPS 1 µg/ml 4h
Lane 7 miR-369-3p mimic 50 nM + LPS 1 µg/ml 4h
Lane 8 Mock + LPS 1µg/ml 4h + Nigericin 20µM 30 min
Lane 9 miR-369-3p mimic 30 nM + LPS 1 µg/ml 4h + Nigericin 20 µM 30 min
Lane 10 miR-369-3p mimic 50 nM + LPS 1 µg/ml 4h + Nigericin 20 µM 30 min

GAPDH

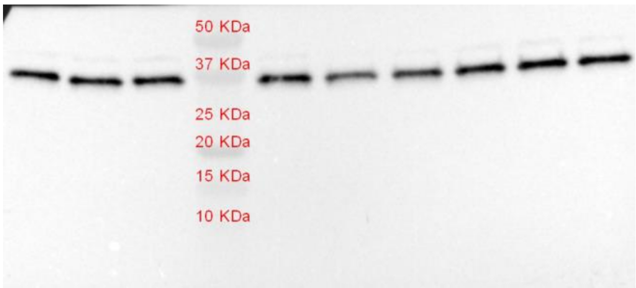

Lane 1 Mock
Lane 2 miR-369-3p mimic 30 nM
Lane 3 miR-369-3p mimic 50 nM
Lane 4 Standard Molecular Weight
Lane 5 Mock + LPS 1 µg/ml 4h
Lane 6 miR-369-3p mimic 30 nM + LPS 1 µg/ml 4h
Lane 7 miR-369-3p mimic 50 nM + LPS 1 µg/ml 4h
Lane 8 Mock + LPS 1µg/ml 4h + Nigericin 20µM 30 min
Lane 9 miR-369-3p mimic 30 nM + LPS 1 µg/ml 4h + Nigericin 20 µM 30 min
Lane 10 miR-369-3p mimic 50 nM + LPS 1 µg/ml 4h + Nigericin 20 µM 30 min

Experiment 3

Caspase-1

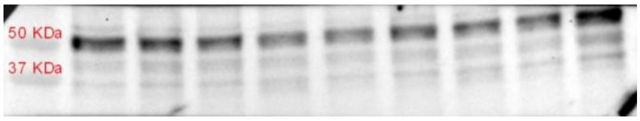

Lane 1 Standard Molecular Weight
Lane 2 Mock
Lane 3 miR-369-3p mimic 30 nM
Lane 4 miR-369-3p mimic 50 nM
Lane 5 Mock + LPS 1 µg/ml 4h
Lane 6 miR-369-3p mimic 30 nM + LPS 1 µg/ml 4h
Lane 7 miR-369-3p mimic 50 nM + LPS 1 µg/ml 4h
Lane 8 Mock + LPS 1 µg/ml 4h + Nigericin 20 µM 30 min
Lane 9 miR-369-3p mimic 30 nM + LPS 1 µg/ml 4h + Nigericin 20 µM 30 min
Lane 10 miR-369-3p mimic 50 nM + LPS 1 µg/ml 4h + Nigericin 20 µM 30 min

GAPDH

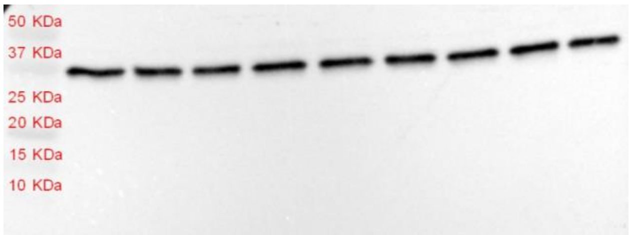

Lane 1 Standard Molecular Weight
Lane 2 Mock
Lane 3 miR-369-3p mimic 30 nM
Lane 4 miR-369-3p mimic 50 nM
Lane 5 Mock + LPS 1 µg/ml 4h
Lane 6 miR-369-3p mimic 30 nM + LPS 1 µg/ml 4h
Lane 7 miR-369-3p mimic 50 nM + LPS 1 µg/ml 4h
Lane 8 Mock + LPS 1 µg/ml 4h + Nigericin 20 µM 30 min
Lane 9 miR-369-3p mimic 30 nM + LPS 1 µg/ml 4h + Nigericin 20 µM 30 min
Lane 10 miR-369-3p mimic 50 nM + LPS 1 µg/ml 4h + Nigericin 20 µM 30 min

Experiment 4

Caspase-1

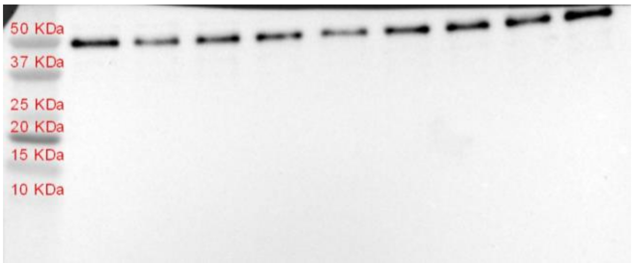

Lane 1 Standard Molecular Weight
Lane 2 Mock
Lane 3 miR-369-3p mimic 30 nM
Lane 4 miR-369-3p mimic 50 nM
Lane 5 Mock + LPS 1 µg/ml 4h
Lane 6 miR-369-3p mimic 30 nM + LPS 1 µg/ml 4h
Lane 7 miR-369-3p mimic 50 nM + LPS 1 µg/ml 4h
Lane 8 Mock + LPS 1 µg/ml 4h + Nigericin 20 µM 30 min
Lane 9 miR-369-3p mimic 30 nM + LPS 1 µg/ml 4h + Nigericin 20 µM 30 min
Lane 10 miR-369-3p mimic 50 nM + LPS 1 µg/ml 4h + Nigericin 20 µM 30 min

GAPDH

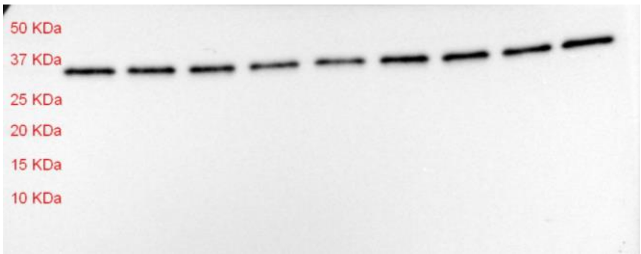

Lane 1 Standard Molecular Weight
Lane 2 Mock
Lane 3 miR-369-3p mimic 30 nM
Lane 4 miR-369-3p mimic 50 nM
Lane 5 Mock + LPS 1 µg/ml 4h
Lane 6 miR-369-3p mimic 30 nM + LPS 1 µg/ml 4h
Lane 7 miR-369-3p mimic 50 nM + LPS 1 µg/ml 4h
Lane 8 Mock + LPS 1 µg/ml 4h + Nigericin 20 µM 30 min
Lane 9 miR-369-3p mimic 30 nM + LPS 1 µg/ml 4h + Nigericin 20 µM 30 min
Lane 10 miR-369-3p mimic 50 nM + LPS 1 µg/ml 4h + Nigericin 20 µM 30 min

Immunoprecipitation for NLRP3

Experiment 1

IP: UB

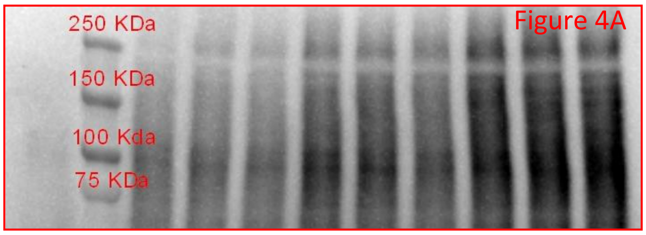

Lane 1 Ab
Lane 2 Standard Molecular Weight
Lane 3 Mock
Lane 4 miR-369-3p mimic 30 nM
Lane 5 miR-369-3p mimic 50 nM
Lane 6 Mock + LPS 1 µg/ml 4h
Lane 7 miR-369-3p mimic 30 nM + LPS 1 µg/ml 4h
Lane 8 miR-369-3p mimic 50 nM + LPS 1 µg/ml 4h
Lane 9 Mock + LPS 1 µg/ml 4h + Nigericin 20 µM 30 min
Lane 10 miR-369-3p mimic 30 nM + LPS 1 µg/ml 4h + Nigericin 20 µM 30 min
Lane 11 miR-369-3p mimic 50 nM + LPS 1 µg/ml 4h + Nigericin 20 µM 30 min

IP:NLRP3

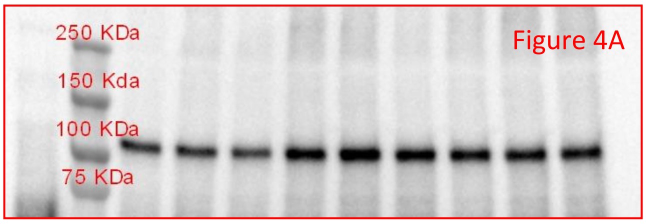

Lane 1 Ab
Lane 2 Standard Molecular Weight
Lane 3 Mock
Lane 4 miR-369-3p mimic 30 nM
Lane 5 miR-369-3p mimic 50 nM
Lane 6 Mock + LPS 1 µg/ml 4h
Lane 7 miR-369-3p mimic 30 nM + LPS 1 µg/ml 4h
Lane 8 miR-369-3p mimic 50 nM + LPS 1 µg/ml 4h
Lane 9 Mock + LPS 1 µg/ml 4h + Nigericin 20 µM 30 min
Lane 10 miR-369-3p mimic 30 nM + LPS 1 µg/ml 4h + Nigericin 20 µM 30 min
Lane 11 miR-369-3p mimic 50 nM + LPS 1 µg/ml 4h + Nigericin 20 µM 30 min

INPUT: BRCC3

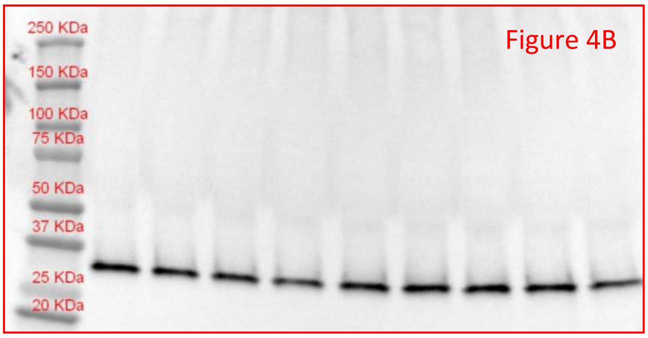

Lane 1 Standard Molecular Weight
Lane 2 Mock
Lane 3 miR-369-3p mimic 30 nM
Lane 4 miR-369-3p mimic 50 nM
Lane 5 Mock + LPS 1 µg/ml 4h
Lane 6 miR-369-3p mimic 30 nM + LPS 1 µg/ml 4h
Lane 7 miR-369-3p mimic 50 nM + LPS 1 µg/ml 4h
Lane 8 Mock + LPS 1 µg/ml 4h + Nigericin 20 µM 30 min
Lane 9 miR-369-3p mimic 30 nM + LPS 1 µg/ml 4h + Nigericin 20 µM 30 min
Lane 10 miR-369-3p mimic 50 nM + LPS 1 µg/ml 4h + Nigericin 20 µM 30 min

INPUT: NLRP3

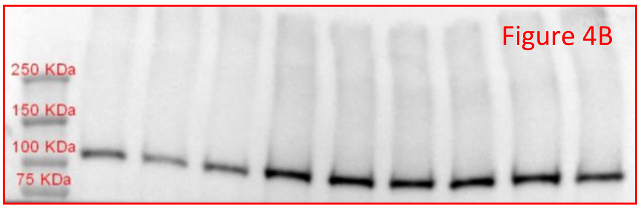

Lane 1 Standard Molecular Weight
Lane 2 Mock
Lane 3 miR-369-3p mimic 30 nM
Lane 4 miR-369-3p mimic 50 nM
Lane 5 Mock + LPS 1 µg/ml 4h
Lane 6 miR-369-3p mimic 30 nM + LPS 1 µg/ml 4h
Lane 7 miR-369-3p mimic 50 nM + LPS 1 µg/ml 4h
Lane 8 Mock + LPS 1 µg/ml 4h + Nigericin 20 µM 30 min
Lane 9 miR-369-3p mimic 30 nM + LPS 1 µg/ml 4h + Nigericin 20 µM 30 min
Lane 10 miR-369-3p mimic 50 nM + LPS 1 µg/ml 4h + Nigericin 20 µM 30 min

INPUT: GAPDH

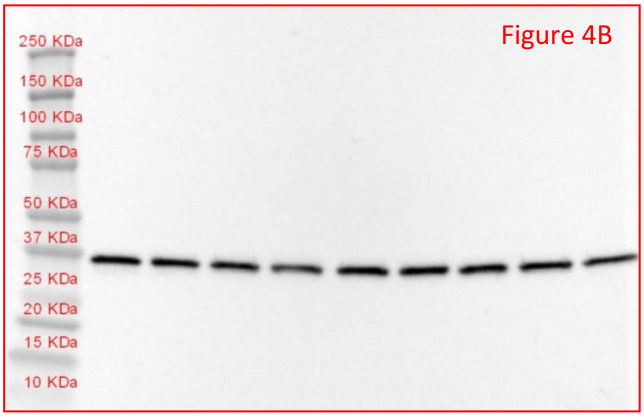

Lane 1 Standard Molecular Weight
Lane 2 Mock
Lane 3 miR-369-3p mimic 30 nM
Lane 4 miR-369-3p mimic 50 nM
Lane 5 Mock + LPS 1 µg/ml 4h
Lane 6 miR-369-3p mimic 30 nM + LPS 1 µg/ml 4h
Lane 7 miR-369-3p mimic 50 nM + LPS 1 µg/ml 4h
Lane 8 Mock + LPS 1 µg/ml 4h + Nigericin 20 µM 30 min
Lane 9 miR-369-3p mimic 30 nM + LPS 1 µg/ml 4h + Nigericin 20 µM 30 min
Lane 10 miR-369-3p mimic 50 nM + LPS 1 µg/ml 4h + Nigericin 20 µM 30 min

Experiment 2

IP: UB

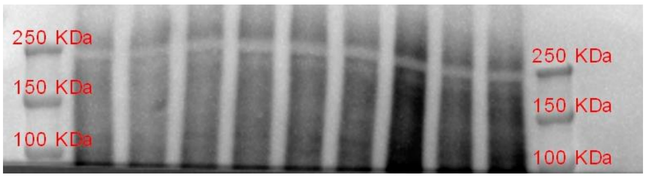

Lane 1 Standard Molecular Weight
Lane 2 Mock
Lane 3 miR-369-3p mimic 30 nM
Lane 4 miR-369-3p mimic 50 nM
Lane 5 Mock + LPS 1 µg/ml 4h
Lane 6 miR-369-3p mimic 30 nM + LPS 1 µg/ml 4h
Lane 7 miR-369-3p mimic 50 nM + LPS 1 µg/ml 4h
Lane 8 Mock + LPS 1 µg/ml 4h + Nigericin 20 µM 30 min
Lane 9 miR-369-3p mimic 30 nM + LPS 1 µg/ml 4h + Nigericin 20 µM 30 min
Lane 10 miR-369-3p mimic 50 nM + LPS 1 µg/ml 4h + Nigericin 20 µM 30 min
Lane 11 Standard Molecular Weight
Lane 12 Ab

IP: NLRP3

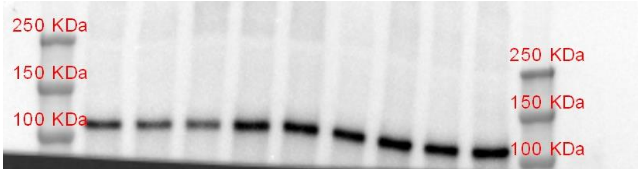

Lane 1 Standard Molecular Weight
Lane 2 Mock
Lane 3 miR-369-3p mimic 30 nM
Lane 4 miR-369-3p mimic 50 nM
Lane 5 Mock + LPS 1 µg/ml 4h
Lane 6 miR-369-3p mimic 30 nM + LPS 1 µg/ml 4h
Lane 7 miR-369-3p mimic 50 nM + LPS 1 µg/ml 4h
Lane 8 Mock + LPS 1 µg/ml 4h + Nigericin 20 µM 30 min
Lane 9 miR-369-3p mimic 30 nM + LPS 1 µg/ml 4h + Nigericin 20 µM 30 min
Lane 10 miR-369-3p mimic 50 nM + LPS 1 µg/ml 4h + Nigericin 20 µM 30 min
Lane 11 Standard Molecular Weight
Lane 12 Ab

INPUT: BRCC3

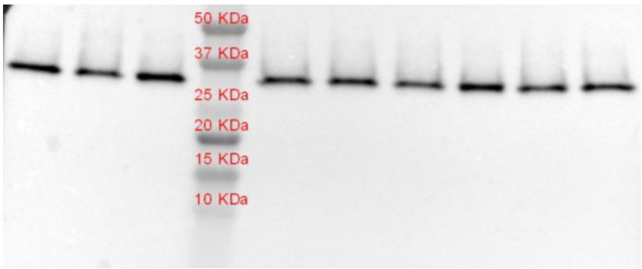

Lane 1 Mock
Lane 2 miR-369-3p mimic 30 nM
Lane 3 miR-369-3p mimic 50 nM
Lane 4 Standard Molecular Weight
Lane 5 Mock + LPS 1 µg/ml 4h
Lane 6 miR-369-3p mimic 30 nM + LPS 1 µg/ml 4h
Lane 7 miR-369-3p mimic 50 nM + LPS 1 µg/ml 4h
Lane 8 Mock + LPS 1µg/ml 4h + Nigericin 20µM 30 min
Lane 9 miR-369-3p mimic 30 nM + LPS 1 µg/ml 4h + Nigericin 20 µM 30 min
Lane 10 miR-369-3p mimic 50 nM + LPS 1 µg/ml 4h + Nigericin 20 µM 30 min

INPUT: NLRP3

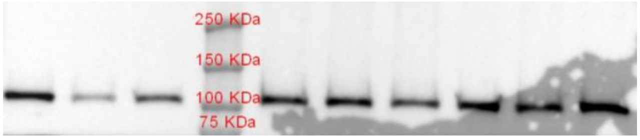

Lane 1 Mock
Lane 2 miR-369-3p mimic 30 nM
Lane 3 miR-369-3p mimic 50 nM
Lane 4 Standard Molecular Weight
Lane 5 Mock + LPS 1 µg/ml 4h
Lane 6 miR-369-3p mimic 30 nM + LPS 1 µg/ml 4h
Lane 7 miR-369-3p mimic 50 nM + LPS 1 µg/ml 4h
Lane 8 Mock + LPS 1µg/ml 4h + Nigericin 20µM 30 min
Lane 9 miR-369-3p mimic 30 nM + LPS 1 µg/ml 4h + Nigericin 20 µM 30 min
Lane 10 miR-369-3p mimic 50 nM + LPS 1 µg/ml 4h + Nigericin 20 µM 30 min

INPUT: GAPDH

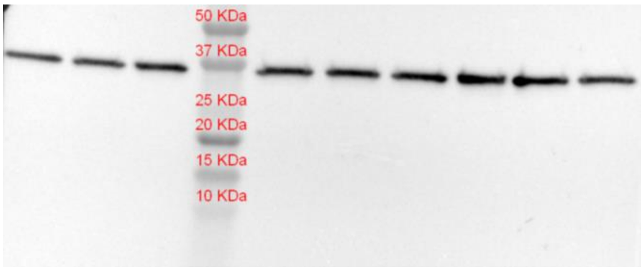

Lane 1 Mock
Lane 2 miR-369-3p mimic 30 nM
Lane 3 miR-369-3p mimic 50 nM
Lane 4 Standard Molecular Weight
Lane 5 Mock + LPS 1 µg/ml 4h
Lane 6 miR-369-3p mimic 30 nM + LPS 1 µg/ml 4h
Lane 7 miR-369-3p mimic 50 nM + LPS 1 µg/ml 4h
Lane 8 Mock + LPS 1µg/ml 4h + Nigericin 20µM 30 min
Lane 9 miR-369-3p mimic 30 nM + LPS 1 µg/ml 4h + Nigericin 20 µM 30 min
Lane 10 miR-369-3p mimic 50 nM + LPS 1 µg/ml 4h + Nigericin 20 µM 30 min

Experiment 3

IP: UB

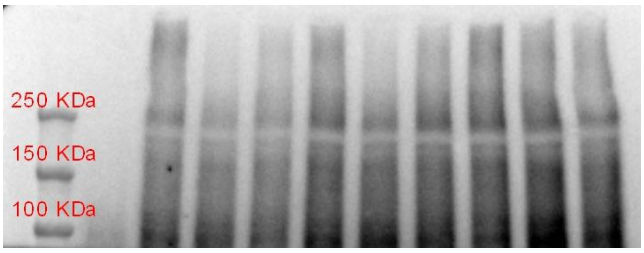

Lane 1 Standard Molecular Weight
Lane 2 Ab
Lane 3 Mock
Lane 4 miR-369-3p mimic 30 nM
Lane 5 miR-369-3p mimic 50 nM
Lane 6 Mock + LPS 1 µg/ml 4h
Lane 7 miR-369-3p mimic 30 nM + LPS 1 µg/ml 4h
Lane 8 miR-369-3p mimic 50 nM + LPS 1 µg/ml 4h
Lane 9 Mock + LPS 1 µg/ml 4h + Nigericin 20 µM 30 min
Lane 10 miR-369-3p mimic 30 nM + LPS 1 µg/ml 4h + Nigericin 20 µM 30 min
Lane 11 miR-369-3p mimic 50 nM + LPS 1 µg/ml 4h + Nigericin 20 µM 30 min

IP: NLRP3

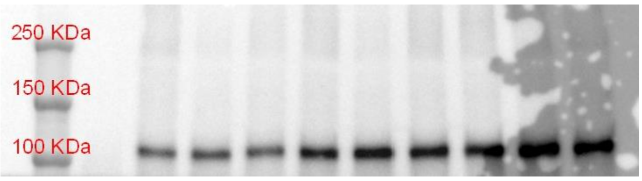

Lane 1 Standard Molecular Weight
Lane 2 Ab
Lane 3 Mock
Lane 4 miR-369-3p mimic 30 nM
Lane 5 miR-369-3p mimic 50 nM
Lane 6 Mock + LPS 1 µg/ml 4h
Lane 7 miR-369-3p mimic 30 nM + LPS 1 µg/ml 4h
Lane 8 miR-369-3p mimic 50 nM + LPS 1 µg/ml 4h
Lane 9 Mock + LPS 1 µg/ml 4h + Nigericin 20 µM 30 min
Lane 10 miR-369-3p mimic 30 nM + LPS 1 µg/ml 4h + Nigericin 20 µM 30 min
Lane 11 miR-369-3p mimic 50 nM + LPS 1 µg/ml 4h + Nigericin 20 µM 30 min

INPUT: BRCC3

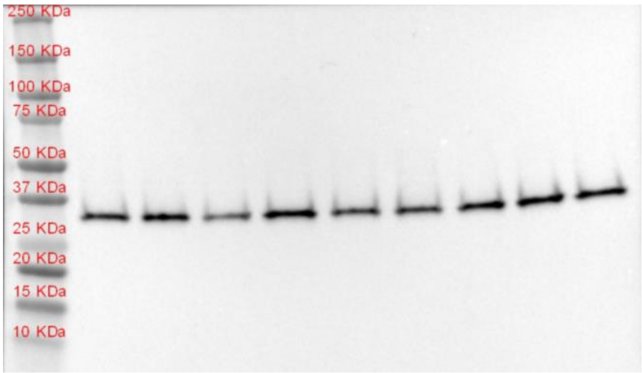

Lane 1 Standard Molecular Weight
Lane 2 Mock
Lane 3 miR-369-3p mimic 30 nM
Lane 4 miR-369-3p mimic 50 nM
Lane 5 Mock + LPS 1 µg/ml 4h
Lane 6 miR-369-3p mimic 30 nM + LPS 1 µg/ml 4h
Lane 7 miR-369-3p mimic 50 nM + LPS 1 µg/ml 4h
Lane 8 Mock + LPS 1 µg/ml 4h + Nigericin 20 µM 30 min
Lane 9 miR-369-3p mimic 30 nM + LPS 1 µg/ml 4h + Nigericin 20 µM 30 min
Lane 10 miR-369-3p mimic 50 nM + LPS 1 µg/ml 4h + Nigericin 20 µM 30 min

INPUT: NLRP3

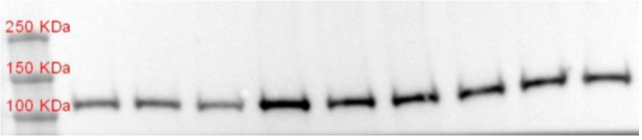

Lane 1 Standard Molecular Weight
Lane 2 Mock
Lane 3 miR-369-3p mimic 30 nM
Lane 4 miR-369-3p mimic 50 nM
Lane 5 Mock + LPS 1 µg/ml 4h
Lane 6 miR-369-3p mimic 30 nM + LPS 1 µg/ml 4h
Lane 7 miR-369-3p mimic 50 nM + LPS 1 µg/ml 4h
Lane 8 Mock + LPS 1 µg/ml 4h + Nigericin 20 µM 30 min
Lane 9 miR-369-3p mimic 30 nM + LPS 1 µg/ml 4h + Nigericin 20 µM 30 min
Lane 10 miR-369-3p mimic 50 nM + LPS 1 µg/ml 4h + Nigericin 20 µM 30 min

INPUT: GAPDH

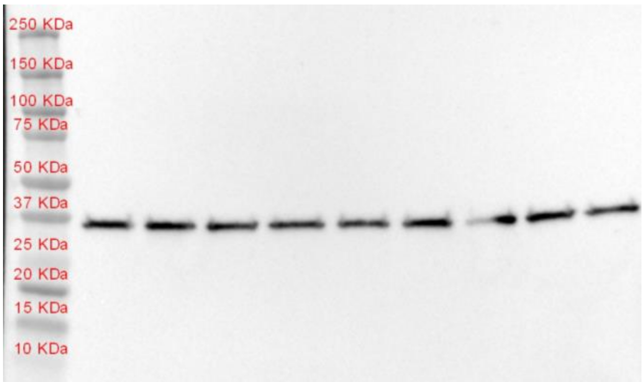

Lane 1 Standard Molecular Weight
Lane 2 Mock
Lane 3 miR-369-3p mimic 30 nM
Lane 4 miR-369-3p mimic 50 nM
Lane 5 Mock + LPS 1 µg/ml 4h
Lane 6 miR-369-3p mimic 30 nM + LPS 1 µg/ml 4h
Lane 7 miR-369-3p mimic 50 nM + LPS 1 µg/ml 4h
Lane 8 Mock + LPS 1 µg/ml 4h + Nigericin 20 µM 30 min
Lane 9 miR-369-3p mimic 30 nM + LPS 1 µg/ml 4h + Nigericin 20 µM 30 min
Lane 10 miR-369-3p mimic 50 nM + LPS 1 µg/ml 4h + Nigericin 20 µM 30 min

Experiment 4

IP: UB

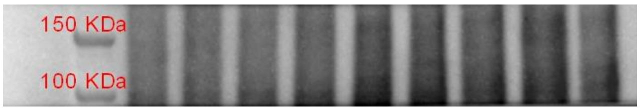

Lane 1 Ab
Lane 2 Standard Molecular Weight
Lane 3 Mock
Lane 4 miR-369-3p mimic 30 nM
Lane 5 miR-369-3p mimic 50 nM
Lane 6 Mock + LPS 1 µg/ml 4h
Lane 7 miR-369-3p mimic 30 nM + LPS 1 µg/ml 4h
Lane 8 miR-369-3p mimic 50 nM + LPS 1 µg/ml 4h
Lane 9 Mock + LPS 1 µg/ml 4h + Nigericin 20 µM 30 min
Lane 10 miR-369-3p mimic 30 nM + LPS 1 µg/ml 4h + Nigericin 20 µM 30 min
Lane 11 miR-369-3p mimic 50 nM + LPS 1 µg/ml 4h + Nigericin 20 µM 30 min

IP: NLRP3

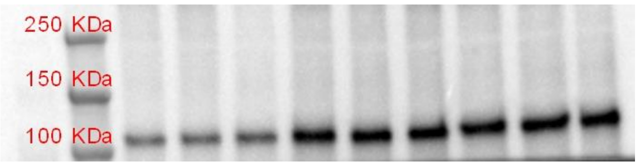

Lane 1 Ab
Lane 2 Standard Molecular Weight
Lane 3 Mock
Lane 4 miR-369-3p mimic 30 nM
Lane 5 miR-369-3p mimic 50 nM
Lane 6 Mock + LPS 1 µg/ml 4h
Lane 7 miR-369-3p mimic 30 nM + LPS 1 µg/ml 4h
Lane 8 miR-369-3p mimic 50 nM + LPS 1 µg/ml 4h
Lane 9 Mock + LPS 1 µg/ml 4h + Nigericin 20 µM 30 min
Lane 10 miR-369-3p mimic 30 nM + LPS 1 µg/ml 4h + Nigericin 20 µM 30 min
Lane 11 miR-369-3p mimic 50 nM + LPS 1 µg/ml 4h + Nigericin 20 µM 30 min

INPUT: BRCC3

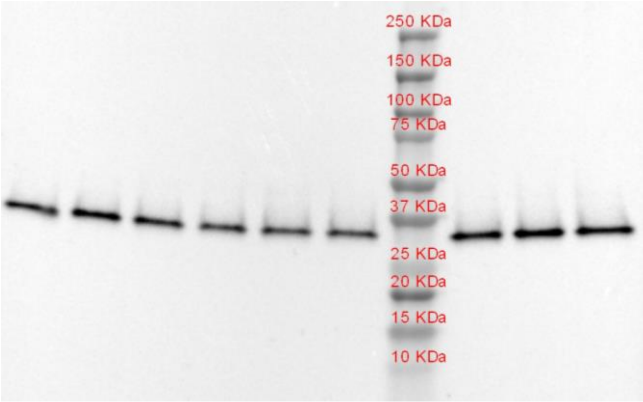

Lane 1 Mock
Lane 2 miR-369-3p mimic 30 nM
Lane 3 miR-369-3p mimic 50 nM
Lane 4 Mock + LPS 1 µg/ml 4h
Lane 5 miR-369-3p mimic 30 nM + LPS 1 µg/ml 4h
Lane 6 miR-369-3p mimic 50 nM + LPS 1 µg/ml 4h
Lane 7 Standard Molecular Weight
Lane 8 Mock + LPS 1 µg/ml 4h + Nigericin 20 µM 30 min
Lane 9 miR-369-3p mimic 30 nM + LPS 1 µg/ml 4h + Nigericin 20 µM 30 min
Lane 10 miR-369-3p mimic 50 nM + LPS 1 µg/ml 4h + Nigericin 20 µM 30 min

INPUT: NLRP3

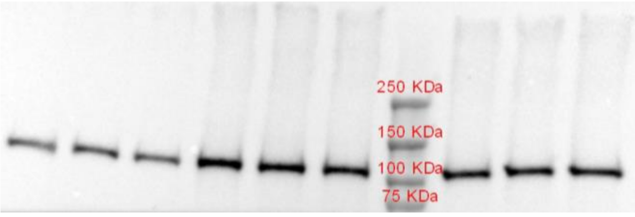

Lane 1 Mock
Lane 2 miR-369-3p mimic 30 nM
Lane 3 miR-369-3p mimic 50 nM
Lane 4 Mock + LPS 1 µg/ml 4h
Lane 5 miR-369-3p mimic 30 nM + LPS 1 µg/ml 4h
Lane 6 miR-369-3p mimic 50 nM + LPS 1 µg/ml 4h
Lane 7 Standard Molecular Weight
Lane 8 Mock + LPS 1 µg/ml 4h + Nigericin 20 µM 30 min
Lane 9 miR-369-3p mimic 30 nM + LPS 1 µg/ml 4h + Nigericin 20 µM 30 min
Lane 10 miR-369-3p mimic 50 nM + LPS 1 µg/ml 4h + Nigericin 20 µM 30 min

INPUT: GAPDH

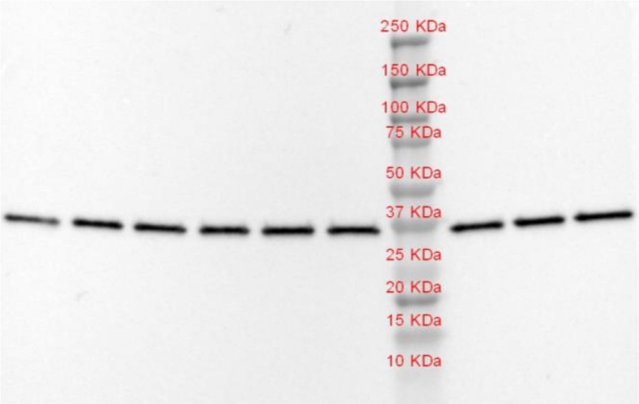

Lane 1 Mock
Lane 2 miR-369-3p mimic 30 nM
Lane 3 miR-369-3p mimic 50 nM
Lane 4 Mock + LPS 1 µg/ml 4h
Lane 5 miR-369-3p mimic 30 nM + LPS 1 µg/ml 4h
Lane 6 miR-369-3p mimic 50 nM + LPS 1 µg/ml 4h
Lane 7 Standard Molecular Weight
Lane 8 Mock + LPS 1 µg/ml 4h + Nigericin 20 µM 30 min
Lane 9 miR-369-3p mimic 30 nM + LPS 1 µg/ml 4h + Nigericin 20 µM 30 min
Lane 10 miR-369-3p mimic 50 nM + LPS 1 µg/ml 4h + Nigericin 20 µM 30 min
